# Impairment of the *trans*-Golgi-Lysosomal Pathway Accelerates Dopaminergic Neuronal Senescence in LRRK2^R1627P^ Rats

**DOI:** 10.14336/AD.2024.0883

**Published:** 2025-10-27

**Authors:** Qiumei Yang, Shimin Pang, Chunsong Zhao, Yanyan Wang, Jing Lu, Zhenyu Yue, Piu Chan

**Affiliations:** ^1^Department of Neurobiology, Neurology and National Clinical Research Center for Geriatric Disorders, Xuanwu Hospital of Capital Medical University, Beijing Institute of Geriatrics, Beijing, China.; ^2^Advanced Innovation Center for Human Brain Protection, Capital Medical University, Beijing, China.; ^3^Clinical Center for Parkinson’s Disease, Capital Medical University, Key Laboratory for Neurodegenerative Disease of the Ministry of Education, Beijing Key Laboratory for Parkinson’s Disease, Beijing, China.; ^4^Beijing Institute of Brain Disorders, Collaborative Innovation Center for Brain Disorders, Capital Medical University, Beijing, China.; ^5^Department of Neurobiology, Icahn School of Medicine at Mount Sinai, New York, USA.

**Keywords:** Parkinson's disease, LRRK2-R1628P mutation, *trans*-golgi-lysosomal pathway, dopaminergic neuronal senescence, gut-brain axis

## Abstract

Leucine-rich repeat kinase 2 (LRRK2)-R1628P mutation has been shown to be one of the common risk factors for Parkinson’s disease (PD) in Asian populations, but the mechanism by which R1628P mutations cause neuronal dysfunction remains unknown. We used LRRK2^R1627P^ knock-in rats (human LRRK2-R1628P corresponds to rat LRRK2-R1627P) to investigate the R1627P mutation on function of dopaminergic neurons (DANs) and their susceptibility to the environmental toxin Lipopolysaccharide (LPS) during aging. LRRK2^R1627P^ rats showed no significant loss of DANs, dopamine and its metabolites, or motor dysfunction; however, spontaneous exploration and olfactory discrimination reduced, and dendritic spines of DANs showed degeneration. We found decreased p^Thr73^-Rab10 located on the *trans*-Golgi, disrupted Golgi structure and lipofuscin accumulation in aged LRRK2^R1627P^ rat DANs, and the protein related to *trans*-Golgi complex and regulating lysosome function were significantly reduced. Although the neuroinflammation of brain was not obvious in the aging process, we confirmed a decrease in the ratio of CD4^+^/CD8^+^ and B cells, an increase in inflammatory factors (TLR4, NFKB, TNF-α) in the periphery. Furthermore, we demonstrated that the R1627P mutation caused the abnormal accumulation of α-Syn in the aged rat intestine. LPS exacerbated pathological α-Syn aggregation in the small intestine of LRRK2 transgenic rats and spread to the brain via the gut-brain axis. This led to microgliosis in the substantia nigra, creating a pro-inflammatory environment and inducing DANs degeneration. Gut-brain axis disruption may be a key determinant of progression to R1628P-PD in R1628P carriers. This insight has important clinical implications and highlights the importance of monitoring and addressing gut-brain axis integrity in individuals with LRRK2 mutations.

## INTRODUCTION

Leucine-rich repeat kinase 2 (LRRK2) mutations are among the most frequent genetic causes of both familial and sporadic Parkinson's disease (PD) [1]. The LRRK2 protein consists of multiple domains, including a GTPase domain and a kinase domain, as well as several protein-protein interaction domains [1]. Pathogenic LRRK2 mutations have been associated with diverse neuropathological outcomes, ranging from pure nigral degeneration without Lewy bodies (LB) to the presence of widespread LB pathology [2-5]. This diversity suggests that different LRRK2 mutations may lead to PD through distinct pathogenic mechanisms.

Among these mutations, the R1628P variant in the COR domain is a common susceptibility allele, particularly in Asian populations. It has been shown to alter LRRK2 kinase activity, converting the adjacent S1627 into a novel phosphorylation site for Cdk5, which upregulates LRRK2 kinase activity and consequently leads to neuronal death [6]. Interestingly, Alexia F Kalogeropulou et al [7]. in their study utilizing immunoblot quantification of 100 LRRK2 variants' kinase activities in HEK293 cell lines, found that the R1628P variant did not stimulate LRRK2 kinase activity. However, when the activity of the R1628P variant was analyzed separately from other activating variants, it was found to significantly inhibit LRRK2 phosphorylation while stimulating Rab10 phosphorylation by approximately two-fold [7]. In vitro analyses of the R1628P variant have produced inconsistent results, thereby obscuring its precise role in the development of PD.

Numerous studies have shown that LRRK2 plays a crucial role in membrane trafficking within the endo-lysosomal pathway [8, 9]. These functions include endocytosis, degradation, and recycling of transmembrane receptors by synaptic vesicles [8, 10], anterograde transport of receptors from the trans-Golgi network to lysosomes [11, 12], particularly through interactions with Rab GTPase proteins [13-15]. Disruption of these pathways has been linked to impaired Golgi-lysosome function [11, 12], which could accelerate dopaminergic neurons (DANs) degeneration and cellular senescence [16, 17]. Despite extensive research on LRRK2 mutations, there is limited understanding of how the R1627P mutation (the rat equivalent of human R1628P) contributes to neuronal dysfunction and neurodegeneration in animal models. To date, there have been no reports on the functional characterization of the R1628P variant in animal models, and it remains unclear whether the disruption of R1628P function caused parkinsonism-like neurodegeneration and loco-motive defects in this model system. In this study, we generated a novel rat model with the R1627P mutation using CRISPR/Cas9 technology and investigated the impact of this mutation on LRRK2 kinase activity. Using this rat model, we examined whether the mutation disrupted normal DANs activity and caused age-related dendritic degeneration. We assessed how the R1627P mutation affected Rab10 phosphorylation and Golgi-lysosome signaling, leading to DANs senescence. In addition, we investigated the immune response in peripheral tissues, including the role of immune imbalance in exacerbating PD pathology through the gut-brain axis. This study aims to elucidate the molecular mechanisms by which the R1627P mutation impairs neuronal health and contributes to PD, providing insights into potential therapeutic targets for addressing neurodegenerative processes.

## Materials and Methods

### Generation of LRRK2^R1627P^ knock-in and LRRK2^-/-^ rats

LRRK2^R1627P^ knock-in and LRRK2^-/-^ Sprague Dawley rats were generated by Biocytogen, Inc. using EGE (CRISPR/Cas9) system methodology. In brief, to generate the gene targeting vector, single guide RNAs (sgRNAs) were designed in the regions of intron 33-34 and intron 34-35 of the LRRK2 gene, respectively, (See details supplemental data 1). Male and female rats were used and analyzed for this study at 2, 8, 16 and 24-26-month-old of age. Rats were kept on a 12 h light/12 h dark cycle, at 20-22 °C, with ad libitum access to food and water.

### Lipopolysaccharide (LPS) administration

LPS (L2880, Escherichia coli O55:B5) was purchased from Sigma-Aldrich (Saint Louis, MO, USA). LPS was intraperitoneally injected into 12-month-old WT, LRRK2^R1627P^ and LRRK2^-/-^ rats (0.1 mg kg^-1^; LPS dissolved in sterile saline), once a week, 6 rats in each group. Rats were sacrificed after 6 months.

### Open-field assay

Open-field measured locomotor activity and rearing [18]. The device is a chamber (Med Associates, Inc.) with a length, width and height of 43.48 cm, 43.48 cm and 30.28cm, respectively, and has three 16-beam Infrared Rays arrays on the X, Y and Z axes for recording horizontal and vertical motion of rats. 2-month-old, 8m-month-old, 16m-month-old and 24-26m-month-old rats were tested. At the start of the test, rats were placed into the arena near the front wall facing left and allowed to freely explore for 15 min. Activity Monitor (Med Associates, Inc.) video tracking software collected rats' motion tracks and analyzed walking distance, walking speed and residence time.

#### Morris water maze assay

Rats were subjected to the Morris water maze task to evaluate learning and memory, as previously reported [19]. 2-month-old, 8-month-old, and 16-month-old rats were tested. In brief, the apparatus was conducted in a round tank, 180cm in diameter and 60cm deep, filled with water. The tank was divided into four equally sized quadrants and a circular acrylic escape platform was placed in one of the quadrants. Rats were placed in different quadrants facing the pool wall at each training and allowed to swim freely until they found and climbed onto the central escape platform. If rats did not locate the platform within 90 seconds, the observer guided them to the platform and allowed them to rest on the platform for 10 seconds. The training lasted for consecutive days (4 trials/day). After completing the five days of training, the platform was removed on the sixth day, and all parameters were recorded semiautomatically by a video tracking system (EthoVision).

### Novel object recognition (NOR) assay

The NOR assay tests for recognition of familiar and novel objects and involves recollection and familiarity [20]. 2-month-old, 8m-month-old, and 16m-month-old rats were tested. Rats were allowed to explore the same arena as in Open-field for 5 min, but containing two objects, placed in opposite corners of one side. The two objects were exactly the same color and shape. Then rats were removed to home cage. After 1 hour, rats were reintroduced to the arena where one of the two objects (familiar objects) was replaced with a shape and color completely distinct from the previous one (novel object), as depicted in Supplementary Fig. 6E and allowed to explore freely for 5 min. The exploration time of rats on familiar objects (F) and novel objects (N) were recorded and calculate the discrimination index (DI) [DI=(N-F)/(N+F)]×100%] and the recognition index (RI) [RI=N/(N+F) ×100%].

### Grip strength assay

Grip strength assay was conducted essentially as described previously [21]. It was assessed by the grasping applied by the rats on a grid that is connected to a sensor. Three trials were carried out in succession measuring forelimb-strength, with an interval of 1h between the two training sessions. 2-month-old,8-month-old and 16-month-old rats were tested. All grip strength values obtained were normalized against rat’s body weight.

### Rotarod assay

Based on the published rotarod assay method, made appropriate modifications to measure the motor coordination, balance and motor learning ability of rats [22]. Rats wereplaced on an accelerating rotarod starting at 4 r.p.m min^-1^. and accelerating to 24 r.p.m min^-1^ within 180s. The time it took for the ratsto fall off the rod was measured. The rats were tested four times a day with a minimal rest period of 30 min between assessments and were tested for either 2 or 3 consecutive days at 2-month.

### Olfaction assay

The olfactory discrimination assay was performed using the habituation/dishabituation test commonly used in rodents [23]. Before the formal experiment, rats explored freely in the behavioral box to familiarize themselves with the environment for 2 days, 10 min/day. Each animal was tested 6 times, with the first 5 tests habituating the rat to a certain odor. For the first 5 tests, 2 porcelain dishes were dripped with lemon essence and distilled water. The rats were allowed to explore freely for 5 min each time, with an interval of 15 min between two tests. In the sixth test, mint essence and distilled water were dripped into the two porcelain dishes. The time was counted when the rat was within 1 cm of the porcelain plate (mint essence) and made an olfactory response. If the residence time of the sixth test is longer than that of the fifth test, it means that the rat can distinguish two different odors. For testing smell sensitivity, binary steps of dilution were presented. A rat responding correctly to a porcelain dish with Mint flavor was tested on the next, lower dilution step. If the animal responded incorrectly it was retested in the previous stronger step to determine the threshold.

### Striatal (Str) and substantia nigra pars compacta (SNc) dopamine measurements by high-performance liquid chromatography (HPLC)

HPLC with electrochemical detection was used to measure the concentration of dopamine and its metabolites in the Str and SNc. Frozen Str and SNc (15 biological replicates per genotype) were sonicated in ice-cold solution (0.1 M perchloric acid/0.2 mM sodium bisulfite) and centrifuged for 10min at 14,000 × g at 4°C. A total of 20 μL of the supernatant was injected onto a C-18 reverse phase Spheri 5, RP-18, 4.6 mm 25 cm catecholamine column (BASi). The mobile phase comprised 0.15 M chloroacetic acid, 0.2 mM EDTA, 0.86 mM sodium octyl sulfate, along with 4% acetonitrile and 2.5% tetrahydrofuran, adjusted to a pH of 3.0. The flow rate was maintained at 1.5 mL/min. Data collection and processing were conducted using Empower 3 software.

### Primary neuronal cell cultures

Primary neuronal cultures from the SNc of postnatal day 0 (P0) pups were prepared as described previously. In brief, neurons were dissociated by pre-warmed (37 ºC) 0.05% trypsin-EDTA (Gibco) and were then placed in the poly-D-lysine coated culture dishes (BD). They were incubated in DMEM/F-12 media, supplemented with 0.5% insulin, 0.5% transferrin, 0.4% putrescine, 0.5 μg/ml Na selenite, 0.635 μg/ml progesterone, 5% FBS and 1% penicillin/streptomycin. Arabinosylcytosine (Sigma) was used to inhibit glial cell growth.

### Fluorescence-activated cell sorting (FACS)

Whole blood was collected via retro-orbital bleedings for FACS analysis. Briefly, cells were washed and stained with Live/Dead dye (eBioscience) in PBS. Cells were blocked with 5% Bovine Serum Albumin (BSA) for 30min. Cells were then stained with antibodies in buffer containing PBS, 1% BSA and 0.1% Sodium Azide at 4°C for 30 mins followed by further washing and cell fixation (BD Cell Fix). See the supplementary information for antibody details.

**Figure 1.**
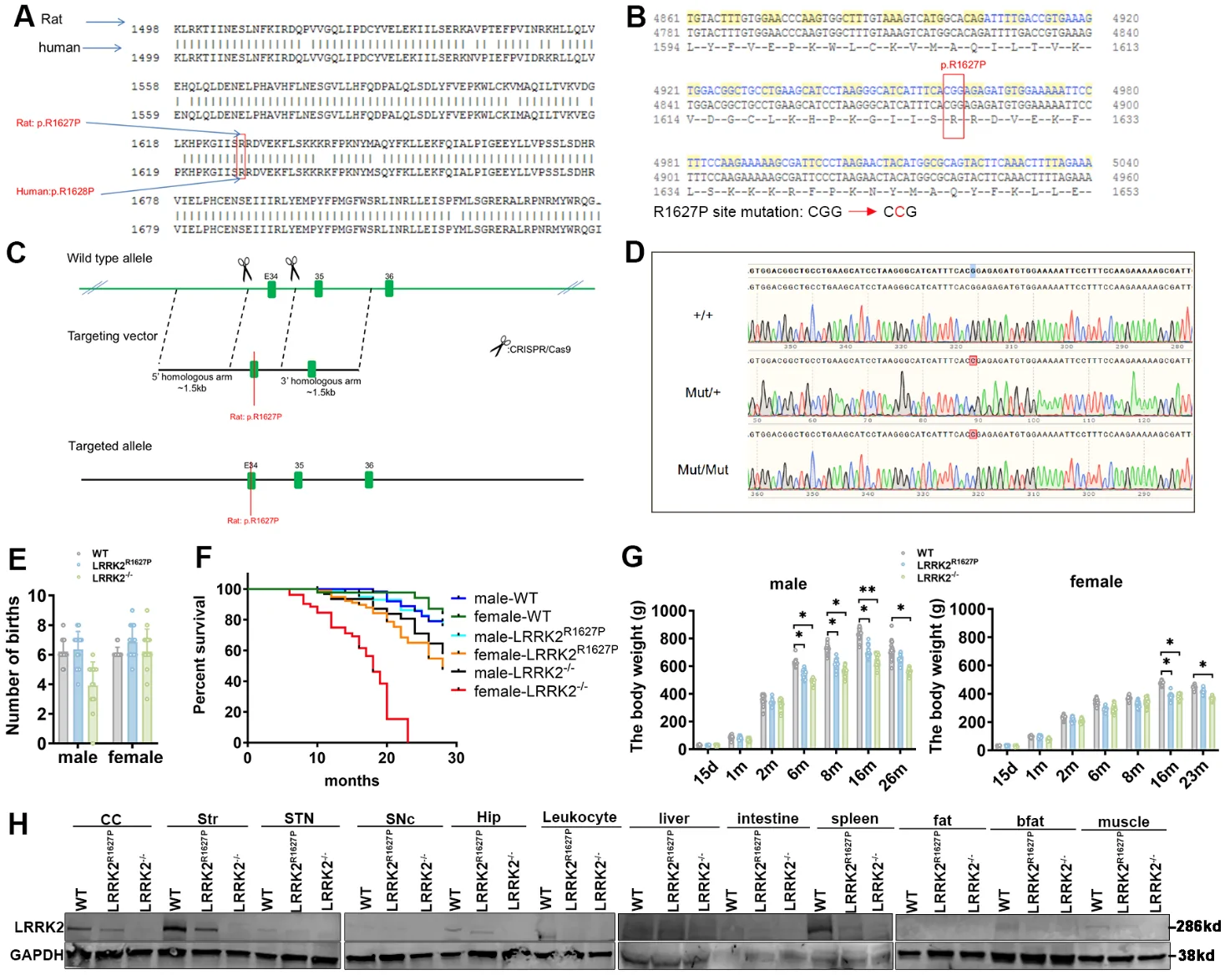
**Generation of LRRK2^R1627P^ and LRRK2^-/-^ rat using CRISPR-Cas9**. (**A**) Partial amino acids sequence alignment between human and mouse; (B) The 1627th amino acid residue Arg within the exon 34 of the Rat LRRK2 gene; (C) Targeting strategy-EGE (CRISPR/Cas9) system to construct LRRK2^R1627P^ KI rat; (D) Sanger sequence verification of the R1627P and silent mutations in the endogenous rat LRRK2 locus; (E) The birth rate of LRRK2^R1627P^ and LRRK2^-/-^ rat (N=30); (F) The mortality rate of LRRK2^R1627P^ and LRRK2^-/-^ rat; (G) The weight of LRRK2^R1627P^ and LRRK2^-/-^ rats of different ages (N=30); (H) The protein expression of LRRK2 in different organizations. Data represent mean ± SD.*P<0.05, **P<0.01.

### Golgi-Cox impregnation of brain tissue and dendritic spine detection

Rats were perfused intracardially with 0.9% saline and then 4% PFA/PBS. The brains were dissected and impregnated using a standard Golgi-Cox solution (1% potassium dichromate, 1% mercuric chloride, and 0.8% potassium chromate), according to a previously described method. The brains immersed in the Golgi-Cox solution were stored at room temperature in the dark for 7 days. Each brain sample was transferred from Golgi-Cox solution to tissue-protectant solution (30% sucrose, 1% polyvinylpyrrolidone, 30% ethylene glycol), and stored at 4°C in dark for 5 days, and sectioned coronally (100μm) using a vibratome. Sections were mounted on gelatinized slides and stained. Dorsolateral Str and SNc Golgi-stained sections were imaged under brightfield illumination on a Leica microscope system and a 100× oil immersion lens. DANs and spiny projection neurons were chosen randomly for quantification from three rats per group. The density of spines and filopodia was analyzed by Fiji ImageJ. For spine size, images were acquired, and the full length and head diameter were measured manually with ImageViewer.

### Transmission electron microscopy (TEM)

Brain tissues (1mm^3^) were fixed in glutaraldehyde (2.5%), post-fixed with 1% osmium tetroxide followed by 1% uranyl acetate, dehydrated them through a graded series of ethanol washes and embedded them in resin. The ultrathin sections stained with uranyl acetate followed by lead citrate and viewed on a JEM-1400plus TEM at 120 kV.

### Statistical analysis

Data are expressed as mean ± standard deviation (SD). The statistical analyses were conducted using Graph Pad Prism 9.5.0 software. The Shapiro-Wilk test was employed for normality assessment. If the data were normal, the *t*-test was used to assess the differences between the two groups, and one-way ANOVA coupled with Tukey correct was used to assess differences among the three or more groups. If the data were not normal, nonparametric test was used to assess the differences between the groups. P < 0.05 was statistically significant.

## RESULTS

### Generation and molecular characterization of LRRK2^R1627P^ and LRRK2^-/-^ rats

Based on the amino acid sequence alignment between human and rat LRRK2, the human LRRK2-R1628P corresponds to the rat LRRK2-R2627P. Using the CRISPR/Cas9 system, we generated LRRK2^R1627P^ rats, where the arginine residue at position 1627 in the rat LRRK2 protein was mutated to proline (Fig. 1A-D). We validated the generated rats using PCR and Southern blot. Heterozygous M/+ rats were mated to obtain homozygous M/M (LRRK2^R1627P^) rats, and the genotypes (M/M, M/+, WT) were determined by DNA sequencing. Additionally, we generated LRRK2 gene knockout rats (LRRK2^-/-^) using the Cas9/sgRNA injection method in fertilized eggs (Supplementary Fig. 1A). LRRK2^R1627P^ and LRRK2^-/-^ rats were viable, fertile, and phenotypically normal (Fig. 1E). However, compared to WT, LRRK2^R1627P^ and LRRK2^-/-^ female rats showed a significantly increased mortality rate after middle age (Fig. 1F). There were no significant differences in body size at birth between LRRK2^R1627P^, LRRK2^-/-^ rats, and their littermate WT rats. However, their body weight was significantly reduced from adulthood (6-month) (Fig. 1G). The qPCR results showed that there were no significant changes in the mRNA levels of LRRK2 in various brain regions of LRRK2^M/+^ and LRRK2^R1627P^ rats compared to their WT littermates (Supplementary Fig. 1B). Interestingly, we confirmed that LRRK2 protein levels were reduced ~2-3 folds in various brain regions of LRRK2^M/+^ and LRRK2^R1627P^ rats (Fig. 1H, Supplementary Fig. 1C). Additionally, LRRK2 protein levels were also significantly decreased in peripheral leukocytes and other organs such as kidney, intestine, and skeletal muscle (Fig. 1H).

### LRRK2 kinase activity in the brain of LRRK2^R1627P^ rats

**Figure 2.**
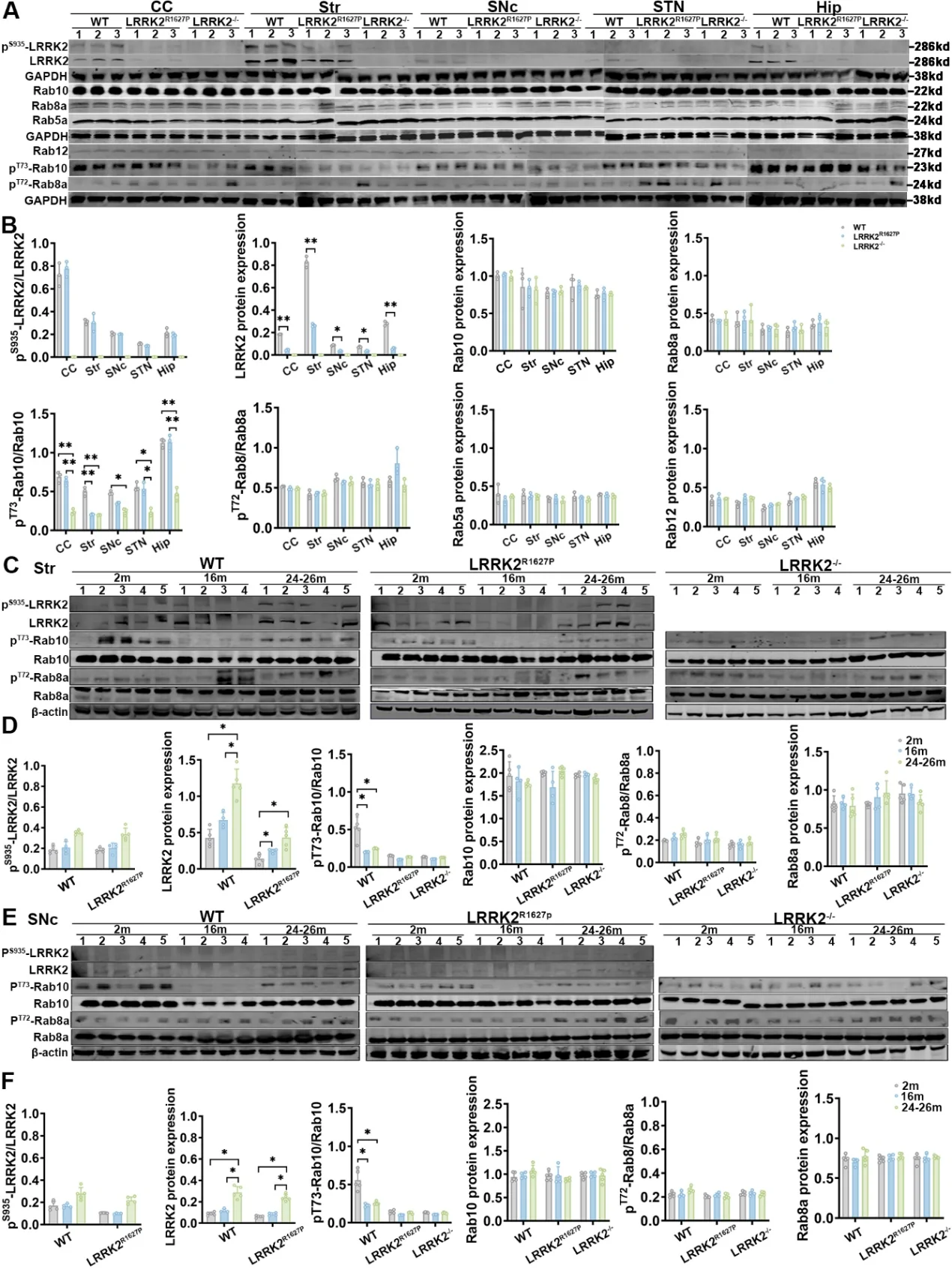
**Age-dependent increase of LRRK2 and steady state levels of LRRK2 phosphorylation in LRRK2^R1627P^ brains**. (A/B) Representative WB and relative quantifications of Ser935 phosphorylation, Thr73-Rab10 phosphorylation, Thr72-Rab8a phosphorylation, total LRRK2 and Rabs (Rab5a, Rab8a, Rab10 and Rab12) in different brain areas of LRRK2^R1627P^ and LRRK2^-/-^ rats (N=3). Quantifications of Ser935 phosphorylation, Thr73-Rab10 phosphorylation and Thr72-Rab8a phosphorylation levels in Str (C/D), SNc (E/F) at 2, 16, and 24-month-old LRRK2^R1627P^ and LRRK2^-/-^ rats (N=4 or 5). Data represent mean ± SD.*P<0.05, **P<0.01.

To explore the LRRK2 kinase activity in the brain of LRRK2^R1627P^ rats, we dissected five brain regions, including the cerebral cortex (CC), Str, subthalamic nucleus (STN), SNc, and hippocampus (Hip), which are widely associated with PD pathology. Phosphorylation of Ser935 reflects LRRK2 activity and is commonly used to assess the engagement of LRRK2 inhibitors [24]. Here, we confirmed that the phosphorylation level of Ser935 showed no significant differences across various brain regions of LRRK2^R1627P^ rats (Fig. 2A, B). Rab GTPases serves as substrates for LRRK2, and LRRK2 regulates various cellular pathways by phosphorylating different Rabs. To understand whether the lack of phospho-Ser935 activation in LRRK2^R1627P^ rat reflects a general inability of this mutation to manifest a similar gain of kinase function observed in cell models [7]. We evaluated the expression levels of Rabs and their phosphorylation. The results showed no significant differences in Rab GTPases, such as Rab5a, Rab8a, Rab10, and Rab12, between LRRK2^R1627P^ and LRRK2^-/-^ rats compared to WT. Notably, p^T73^-Rab10 expression was significantly reduced in LRRK2^-/-^ rats, while it was only decreased in the Str and SNc of LRRK2^R1627P^ rats (Fig. 2A, B). Next, we compared the phosphorylation levels of LRRK2-Ser935 and Rab10-Thr73 in the Str and SNc at different ages. Our results revealed the following: (1) LRRK2 expression increased upon aging both in WT and LRRK2^R1627P^ rats, but the phosphorylation level of Ser935 remained unchanged; (2) p^T73^-Rab10 expression decreased with aging in WT rats, with no further changes observed after 16-month until old age (26-month). However, there were no significant changes in p^T73^-Rab10 expression in LRRK2^R1627P^ and LRRK2^-/-^ rats (Fig. 2C-F).

**Figure 3.**
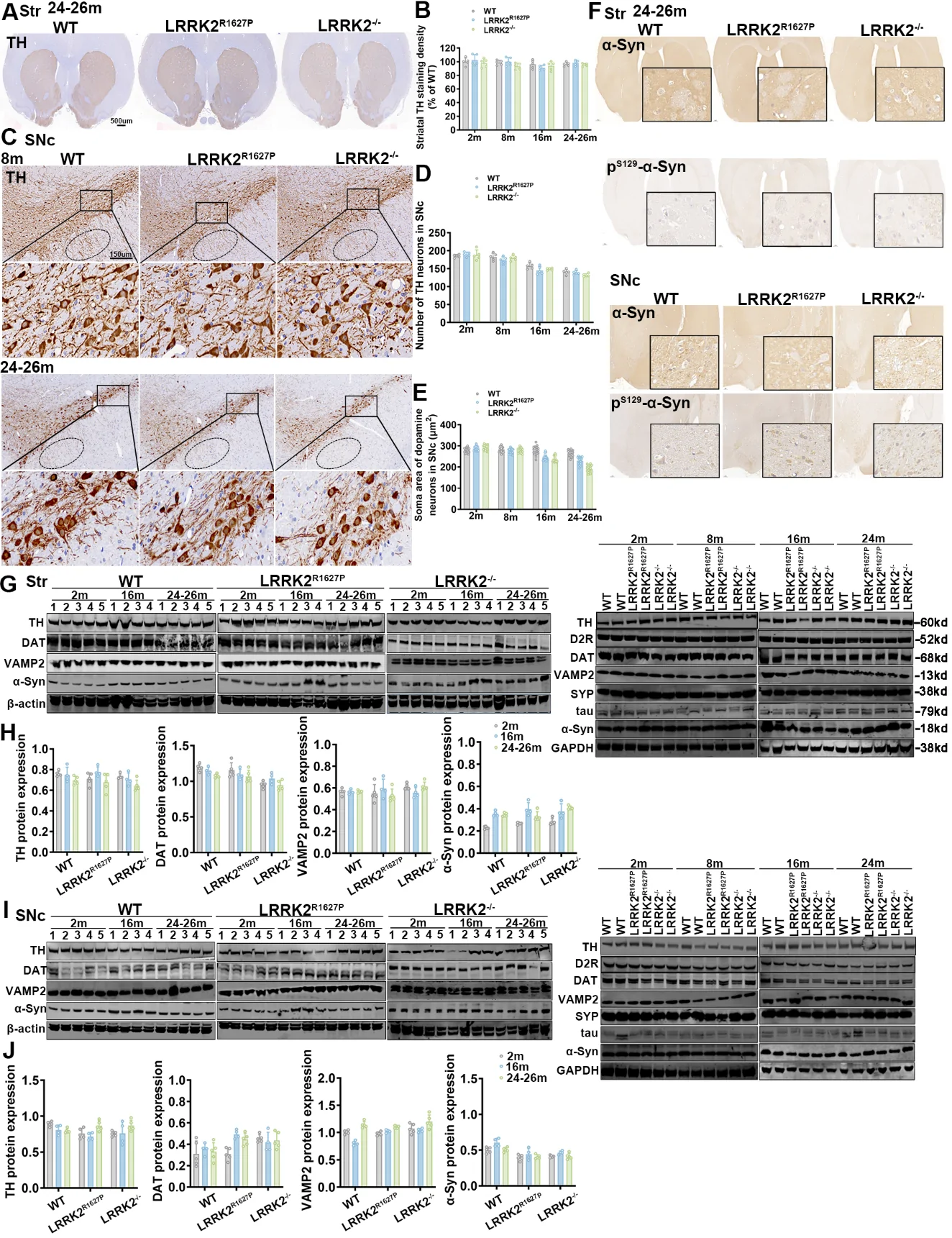
**The R1627P mutation in rats does not cause loss of DANs**. (A, C) No apparent difference in the expression pattern of TH in Str and SNc at 24-26-month-old between WT and LRRK2^R1627P^ or LRRK2^-/-^ rats (N = 5). Scale bar: 500μm in (A) and 150μm in (C). (B, D) There were no statistically significant differences in intensity or number of staining between TH^+^ DANs at Str (B) and SNc (D) (N = 5). (**E**) Statistical analysis of soma area of TH^+^ DANs in the SNc of different ages (N = 5). (**F**) No apparent difference in the expression patterns of α-Syn and p^S129^-α-Syn in Str and SNc between WT LRRK2^R1627P^ or LRRK2^-/-^ rats at 24-26 months (N = 5). (**G-J**) WB showed similar expression level of TH, D2R, DAT, VAMP, SYP, tau and α-Syn in Str (G/H) and SNc (I/J) between WT and LRRK2^R1627P^ or LRRK2^-/-^ rats at different ages (N=4 or 5). Data represent mean ± SD. *P<0.05, **P<0.01.

**Figure 4.**
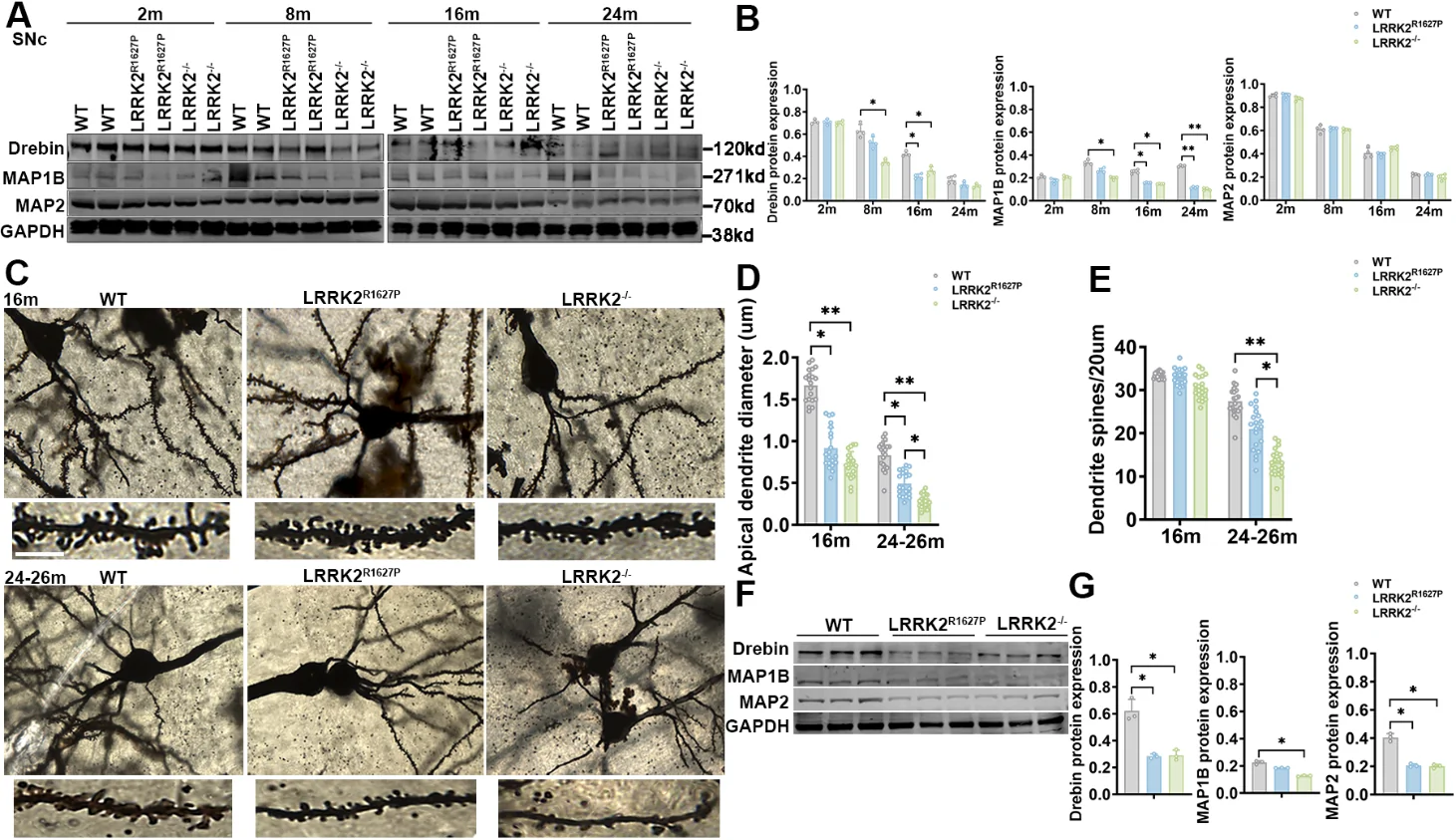
**Loss of LRRK2 leads to reduced dendritic complexity in the SNc at 16-month-old rats**. (A, B) Dendrite-associated protein (Drebin, MAP1B and MAP2) expression in the SNc of WT, LRRK2^R1627P^ and LRRK2^-/-^ rats at different ages (N=4). (**C**) Golgi Cox staining of neurons from the SNc of WT, LRRK2^R1627P^ and LRRK2^-/-^ rats at 16 and 24-26-month-old. The bottom panel is a magnification of the boxed area of the upper panel. Scale bar: 5μm. The apical dendrite diameter (D) and density of dendrite spines (E) (N=3). (F, G) The expression of dendrite-associated proteins (Drebin, MAP1B, and MAP2) in primary cultured WT, LRRK2^R1627P^, and LRRK2^-/-^ rat DANs (N=3). Data represent mean ± SD.*P<0.05, **P<0.01.

### Normal DANs and dopamine levels in LRRK2^R1627P^ rats

To determine whether LRRK2^R1627P^ rat develop degeneration of DANs, we performed immune-histochemical analysis of the LRRK2^R1627P^ rat brains using an antibody specific for the rate-limiting enzyme in dopamine synthesis (TH). The morphology of DANs and their projections appears grossly normal in LRRK2^R1627P^ and LRRK2^-/-^ rats (Fig. 3A-C, S2A-C). Quantification of TH^+^ neurons in SNc using stereological methods revealed no significant difference in the number of DANs between LRRK2^R1627P^ and LRRK2^-/-^ rats and WT littermate at 2 to 24-26-month (Fig. 3D, Supplementary Fig. 2D). However, we observed a significant reduction in distal dendrites of DANs in LRRK2^R1627P^ and LRRK2^-/-^ rats (Fig. 3C, Supplementary Fig. 2C). The soma size of DANs in LRRK2^R1627P^ and LRRK2^-/-^ male rats showed no significant differences compared to WT rats, but in 24-26-month female LRRK2^R1627P^ rats, the soma area of neurons was significantly reduced (Fig. 3C, E, Supplementary Fig. 2C, E). These results reveal the typical age-related decline of midbrain DANs in both LRRK2^R1627P^ and LRRK2^-/-^ rats. Next, we examined DANs axonal neurochemistry by HPLC analysis of total dopamine content in Str and SNc lysates from LRRK2^R1627P^, LRRK2^-/-^ and WT rats at different ages, and found that the levels of dopamine and its metabolites, including: 3,4-dihydroxyphenylacetic-acid (DOPAC), 5-hydroxyindoleacetic-acid (5-HIAA), 5-hydro-ytrypt-amine (5-HT), and homovanillic-acid (HVA), and norepinephrine (NE), were equivalent among LRRK2^R1627P^, LRRK2^-/-^ and WT rats (Supplementary Fig. 3, 4).

Besides the loss of midbrain nigral DANs, the presence of intraneuronal aggregates such as LB is also a hallmark of PD. For patients with LRRK2-linked PD, cases with and without LB pathology have been reported [25]. However, we could not detect α-Syn-positive inclusions or altered expression levels of p^S129^-α-Syn in LRRK2^R1627P^ and LRRK2^-/-^ rats (Fig. 3F). Next, we tested the expression levels of dopamine transporter (DAT), dopamine receptor protein (D2R), protein involved in neurotransmitter release (VAMP, SYP) and tau in LRRK2^R1627P^ and LRRK2^-/-^ rats at different ages, but could not see distinct accumulations or a significant decrease in the level of immunoreactivity in both lines (Fig. 3G-J).

**Figure 5.**
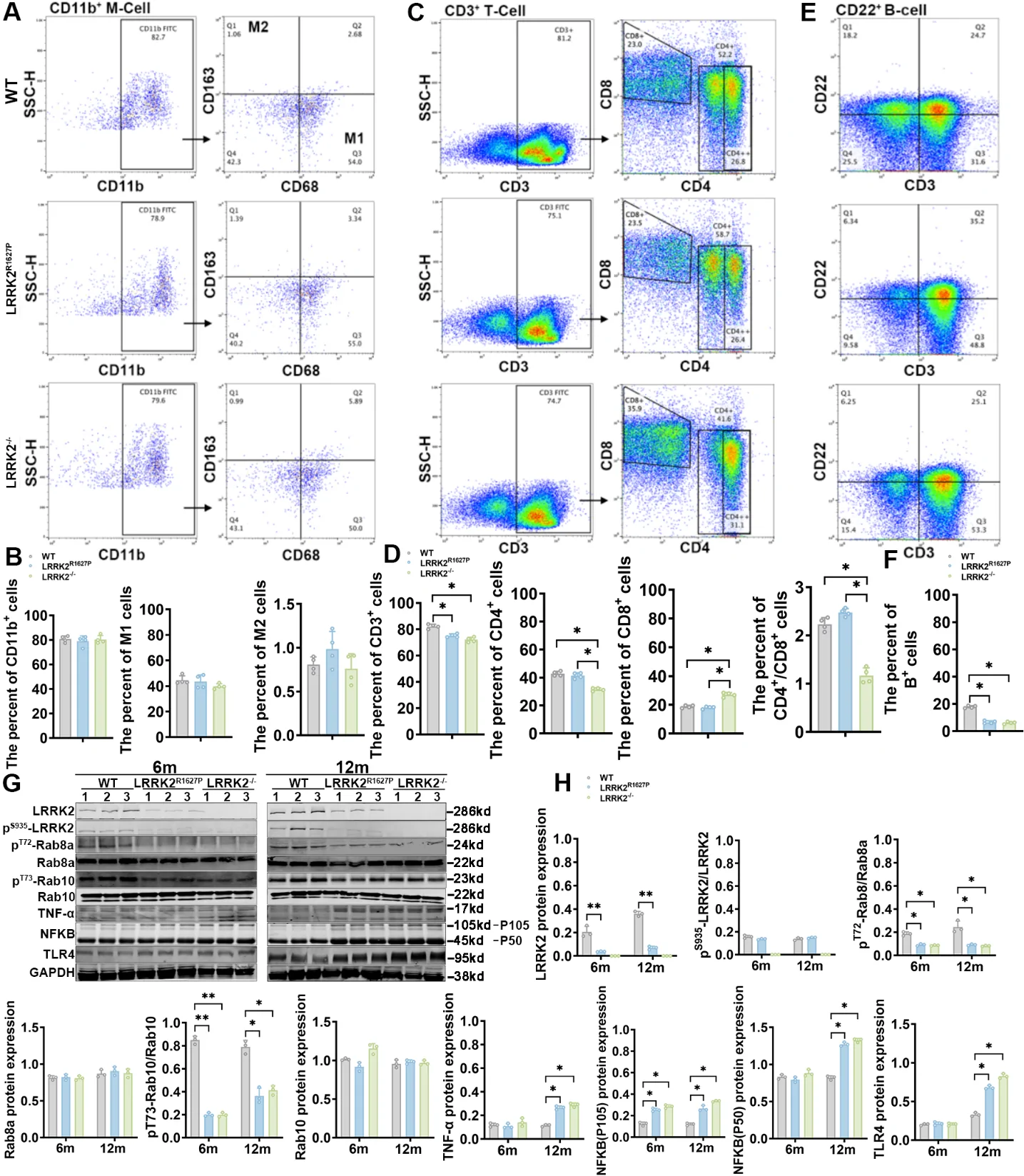
**Increased inflammation in peripheral leukocytes of LRRK2^R1627P^ and LRRK2^-/-^ rats**. Representative flow cytometry plots from leukocytes of WT, LRRK2^R1627P^ and LRRK2^-/-^ rats at 6-month-old, showing numbers of macrophages (A, B), lymphocytes (C, D) and B cells (E, F) (N=4). (G, H) WB showed the protein expression of LRRK2, p^S935^-LRRK2, Rab8a, p^T72^-Rab8a, Rab10, p^T73^-Rab10, TNF-α, NFKB and TLR4 in leukocytes from WT, LRRK2^R1627P^, and LRRK2^-/-^ rats at 6 and 12-month (N=3). Data represent mean ± SD.*P<0.05, **P<0.01.

### Density and morphology of dendritic spines in DANs of SNc from LRRK2^R1627P^ and LRRK2^-/-^ rats

Next, we compared the protein levels of neuronal dendritic markers (drebrin, MAP2, MAP1B) in the SNc of different ages. The results showed a significant reduction in drebrin and MAP1B protein levels in LRRK2^R1627P^ and LRRK2^-/-^ rats at 8-month, compared to the WT group (Fig. 4A, B). For this, the brain of LRRK2^R1627P^ and LRRK2^-/-^ rats and their corresponding WT littermates were stained using the classical technique of Golgi. The morphology of neurons in LRRK2^R1627P^ and LRRK2^-/-^ rats resembled that of the control group. However, it has to be mentioned that the diameter of distal dendritic spines in neurons of LRRK2^R1627P^ and LRRK2^-/-^ rats was significantly reduced at 16-month. Furthermore, the density of dendritic spines showed a significant decrease at 24-26-months (Fig. 4C-E). In addition, further testing was conducted on primary cultures of DANs in vitro to assess the expression levels of drebrin, MAP2, and MAP1B. The results revealed a significant reduction in their expression in LRRK2^R1627P^ and LRRK2^-/-^ rats (Fig. 4F, G).

**Figure 6.**
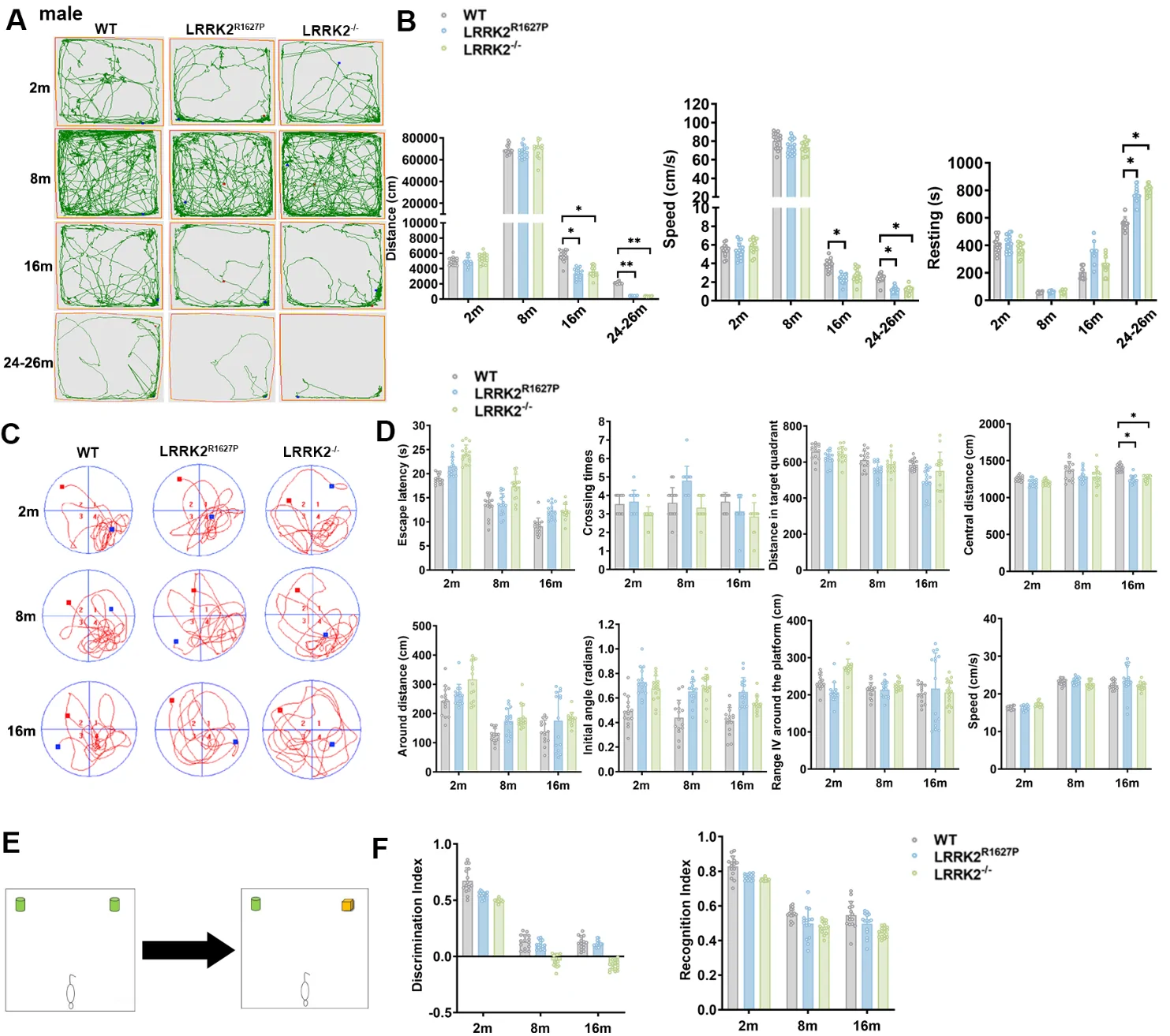
**Behavioral analysis of LRRK2^R1627P^ and LRRK2^-/-^ rats**. (A, B) Open field assay was used to detect the exploratory behavior of WT, LRRK2^R1627P^ and LRRK2^-/-^ rats at different ages (N=16). (C, D) Water maze assay was used to detect the exploratory behavior of WT, LRRK2^R1627P^ and LRRK2^-/-^ rats at different ages. (E, F) The novel object recognition assay was used to detect the exploratory behavior of WT, LRRK2^R1627P^ and LRRK2^-/-^ rats at different ages (N=15). Data represent mean ± SD.*P<0.05, **P<0.01.

**Figure 7.**
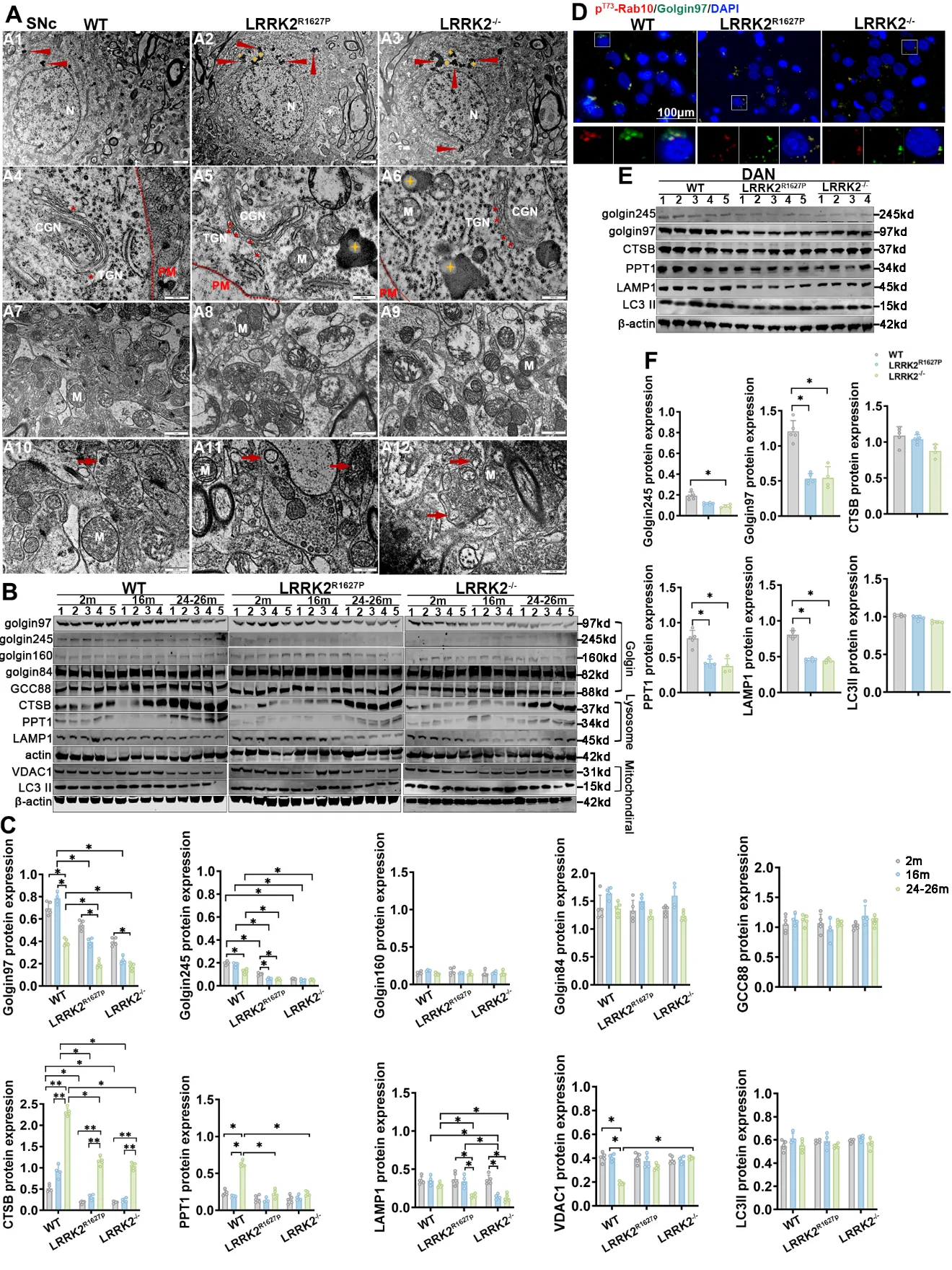
**Age-dependent disruption of Golgi structure in DANs of LRRK2^R1627P^ and LRRK2^-/-^ rats**. (**A**) Electron micrographs of the Golgi apparatus and mitochondrial in DANs from SNc of WT, LRRK2^R1627P^ and LRRK2^-/-^ rats at 24-26-month-old (N=3). (A1-A3) N: nucleus; red head arrow: autolysosomes; yellow asterisks: lipofuscin; bar: 2μm. (A4-A6) CGN: *cis*-Golgi network; TGN: *trans*-Golgi network; PM: plasma membrane; M: mitochondrial; red asterisks: vesicular profiles localized to the *trans*-Golgi; yellow asterisks: lipofuscin; bar: 500nm. (A7-A9) M: mitochondrial; bar: 1μm. (A10-A12) red arrow: cisternae; bar: 500nm. (B, C) WB showed the protein expression of the Golgi apparatus (Golgin-97, Golgin-245, Golgin-84, Golgin-130, and GCC88), lysosomal function (CTSB, PPT1 and LAMP1), and mitochondrial autophagy (VDAC1, LC-II) in SNc of WT, LRRK2^R1627P^, and LRRK2^-/-^ rats at different ages (N=4 or 5). (**D**) Immunofluorescence of DANs for Golgin-97 (green) and pT73-Rab10 (red) in WT, LRRK2^R1627P^, and LRRK2^-/-^ rats (N=4). Scar bar: 200 μm. (E, F) The expression of Golgi apparatus (Golgin-97, Golgin-245), lysosomal function (CTSB, PPT1 and LAMP1), and mitochondrial autophagy (LC-II) in primary cultured WT, LRRK2^R1627P^, and LRRK2^-/-^ rat DANs (N=4 or 5). Data represent mean ± SD.*P<0.05, **P<0.01.

### Peripheral inflammatory profile is altered by mutant R1627P, not in brain

Microglia, a key driving factor of inflammation in the brain. Immunohistochemical analysis was performed to assess the activation state of microglia in the Str and SNc of LRRK2^R1627P^, LRRK2^-/-^, and WT rats. The results revealed no significant changes in the number and morphology of IBA-1^+^ microglia (Supplementary Fig. 5A). This suggests that the R1627P mutation or endogenous LRRK2 deficiency in rats does not activate microglia in the brain. We investigated whether mutant LRRK2 alters the neuroinflammatory profile of the brain further. Thus, we assessed the expression of pro-apoptotic factors (NFKB, TNF-α, IFN-γ), pro-inflammatory factor (TLR4), anti-inflammatory cytokine (TGF-β), and chemokine (CXCL1) in Str and SNc. Similarly, no significant differences were observed in the expression levels of these cytokines in LRRK2^R1627P^, LRRK2^-/-^ and WT rats (Supplementary Fig. 5B/C).

Based on the above experiments showing that LRRK2 mutations do not activate microglia in the brain and lack evidence of bone marrow and T cell infiltration. As LRRK2 is highly express in the peripheral leucocyte, we first observed a significant decrease in p^T72^-Rab8a and p^T73^-Rab10 expression in the periphery of LRRK2^R1627P^ and LRRK2^-/-^ rats, while the expression of pro-apoptotic (NFKB, TNF-α) and pro-inflammatory (TLR4) factors were significantly increased (Fig. 5G, H). Next, we carried out flow cytometric analysis to characterize peripheral mononuclear cell subpopulations in LRRK2^R1627P^, LRRK2^-/-^ and WT rats. Although we observed no significant difference in the number and subpopulation of macrophages (Fig. 5A, B), CD4^+^ T cells were significantly decreased in LRRK2^-/-^ rats, while CD8^+^ T cells were increased. Additionally, the ratio of CD4^+^/CD8^+^ as an immune regulatory marker was significantly decreased in LRRK2^-/-^ rats, but not LRRK2^R1627P^ rats at 6-month (Fig. 5C, D). Furthermore, B cells were also significantly decreased in LRRK2^R1627P^ and LRRK2^-/-^ rats (Fig. 5E, F).

### Analysis of Parkinson's prodromal behavior in LRRK2^R1627P^ and LRRK2^-/-^ rats

#### Motor behavior

Since PD is a progressive age-related disorder, we performed a preliminary analysis of spontaneous exploration data collected in an open field. The evaluation was done in a 15 min monitoring session at each age to minimize the impact of repeated testing and habituation. It showed that no differences in walking distance, walking speed or onset of exploration between the three lines before 8-month-old. At 16-month, both male and female LRRK2^R1627P^ and LRRK2^-/-^ rats had significantly reduced walking distance and walking speed, and they explored the center of the open-field arena significantly less than the WT. Up to 24-26-month, LRRK2^R1627P^ and LRRK2^-/-^ rats were nearly immobile, with occasional exploratory movements only in the corners of the open-field (Fig. 6A/B, Supplementary Fig. 6A, B).

In order to exclude any effect of muscle strength on these behaviors, we also performed the Grip Strength test at different ages, which did not reveal any differences in either rat line (Supplementary Fig. 7A). To further detect subtle changes in locomotion, we chose more sophisticated tests such as swimming and rotarod, and did not reveal any differences with regard to coordination among the three rat lines (Supplementary Fig. 7B, C).

#### Spatial learning and memory

All rats were additionally tested on the Morris water maze in order to assess spatial learning. Based on Fig. 6C, D, male rats of all genotypes at different ages had no significant difference of escape latency, crossing times, distance in target quadrant, speed, residence time in the quadrant of the platform and initial angle. Additionally, we observed no significant changes in speed and distance at 16-month LRRK2^R1627P^ and LRRK2^-/-^ female rats compared with WT, however, their escape latency and distance in the surrounding exploration were significantly increased, while platform crossing times and central distance were decreased (Supplementary Fig. 6C, D). In summary, there were no cognitive and memory deficits detectable in male LRRK2 transgenic rats with the Morris water maze, while spatial learning was impaired in female LRRK2 transgenic rats.

In parallel, we tested the rat in the classic NOR task to compare the performance of these two different methods. The NOR test relies on the natural propensity of rodents to explore novel objects, and it has been used to assess short-term spatial memory [26]. We found that LRRK2^R1627P^ and LRRK2^-/-^ male rats showed no significant changes in discrimination index and recognition index compared to the WT (Fig. 6E, F), indicating that the exploration and learning abilities of these two lines of male rats were normal. But LRRK2^-/-^ female rats exhibited significantly worse scores on the discrimination index and recognition index after 8-month-old (Supplementary Fig. 6E, F).

#### Olfaction

Hyposmia is one of the most widespread non-motor symptoms occurring very early in the course of the disease and it is actually already used for prediction and diagnosis of PD [27]. Based on this, we utilized an odor discrimination paradigm to test olfactory abilities. To test for sensitivity rats had to detect an assigned odor with increasing dilutions. For discrimination abilities they had to discriminate of two different odors [28]. In 24-26-month LRRK2^R1627P^ and LRRK2^-/-^ rats, a significant decrease in olfactory sensitivity and discrimination ability was detected (Supplementary Fig. 8A-C).

### R1627P mutation induces age-dependent Golgi apparatus impairment and lipofuscin buildup in DANs

In the results of Fig. 3 and 4, we observed age-dependent degeneration of dendrites and dendritic spines in DANs in LRRK2^R1627P^ and LRRK2^-/-^ rats (Fig. 3C, 4A). Previous studies have reported that the small GTPase Rab10 was a key regulatory factor in membrane transport during dendritic morphogenesis [29]. Phosphorylated Rab10 interacts with various effector proteins to control post-Golgi vesicle transport [30]. To determine whether the LRRK2^R1627P^-mediated reduction of endogenous p^T73^-Rab10 disrupts Golgi transport and leads to DANs degeneration. We performed TEM analysis on neurons in SNc of LRRK2^R1627P^, LRRK2^-/-^ and WT rats. There was no significant difference in the number of electrondense vesicles between neurons of 16-month LRRK2^R1627P^, LRRK2^-/-^, and WT rats (data not shown). However, there was a significant increase in LRRK2^R1627P^ and LRRK2^-/-^ neurons at 24-26 month (Fig. 7A1-A3). Higher-magnification views further revealed the presence of autophagosomes and autolysosomes in 24-26-month LRRK2^R1627P^ and LRRK2^-/-^ neurons, as well as large electrondense lipofuscin-like granules composed of lipid components defined as round lipofuscin vacuoles (Fig 7A4-A6). Additionally, we observed loss of Golgi ribbon and formation of "mini stacks", as well as accumulation of vesicles in the *trans*-Golgi region in LRRK2^R1627P^ and LRRK2^-/-^ rat neurons (Fig. 7A4-A6, 7D), indicating that the reduction of endogenous p^T73^-Rab10 mediated by the R1627P mutation in rats disrupted Golgi structure. Next, we identified the protein expression of Golgin160 (*cis*-Golgi), Golgin84 (Golgi stack margins), Golgin97, Golgin245 and GCC88 (*trans*-Golgi) in SNc at different ages. The results showed a significant decrease in Golgin97 and Golgin245 in SNc of LRRK2^R1627P^ and LRRK2^-/-^ rats, which decreased with age in all genotypes. However, the expressions of GCC88, Golgin84, and Golgin160 did not show significant changes either among different genotypes of rats or with age (Fig. 7B, C). Correspondingly, we observed a significant age-dependent decrease in the expression of proteins involved in lysosomal function, such as CTSB, PPT1, and LAMP1, in SNc of LRRK2^R1627P^ and LRRK2^-/-^ rats, while the expression of LC3 II remained unchanged (Fig. 7B, C). To further determine the impact of the R1627P mutation or endogenous LRRK2 deficiency on the Golgi-lysosome signaling function in DANs, we confirmed that Golgin97, PPT1, and LAMP1 were significantly reduced in DANs derived from the SNc of LRRK2^R1627P^, LRRK2^-/-^ and WT rats. However, the expressions of Golgin245, CTSB, and LC3 II did not show any significant changes (Fig. 7E, F).

### LPS exacerbates pathological α-Syn accumulation in the small intestine of LRRK2^R1627P^ rats and spreads to the brain via the gut-brain axis

Intestinal inflammation and pathological α-Syn deposition, which can occur before the onset of PD motor symptoms, have been identified as important gastrointestinal features of PD. As a common risk gene for PD and inflammatory bowel disease (IBD), LRRK2 suggests a close connection between the brain and intestines. Therefore, we also observed pathophysiological changes in the intestine of LRRK2 transgenic rats throughout their life cycle. HE staining results showed that with increasing age, the villus height and crypt depth of the small intestine of 8-month-old LRRK2 transgenic rats decreased compared with WT rats (Supplementary Fig. 9A); However, until the old age, the intestines of the three groups of rats (WT, LRRK2^R1627P^ and LRRK2^-/-^ rats) did not show obvious pathological changes such as edema, bleeding, and ulcers. Unexpectedly, we found that the expression of α-Syn and p^S129^-α-Syn increased significantly in the small intestine of LRRK2 transgenic rats at 16-months-old, and this change was mainly concentrated in the lamina propria of the small intestine (Supplementary Fig. 9B-E). It is noteworthy that age-dependent α-Syn abnormal expression in rats caused by R1627P mutation or LRRK2^-/-^ occurs only in the intestine, and this change is not observed in the brain.

**Figure 8.**
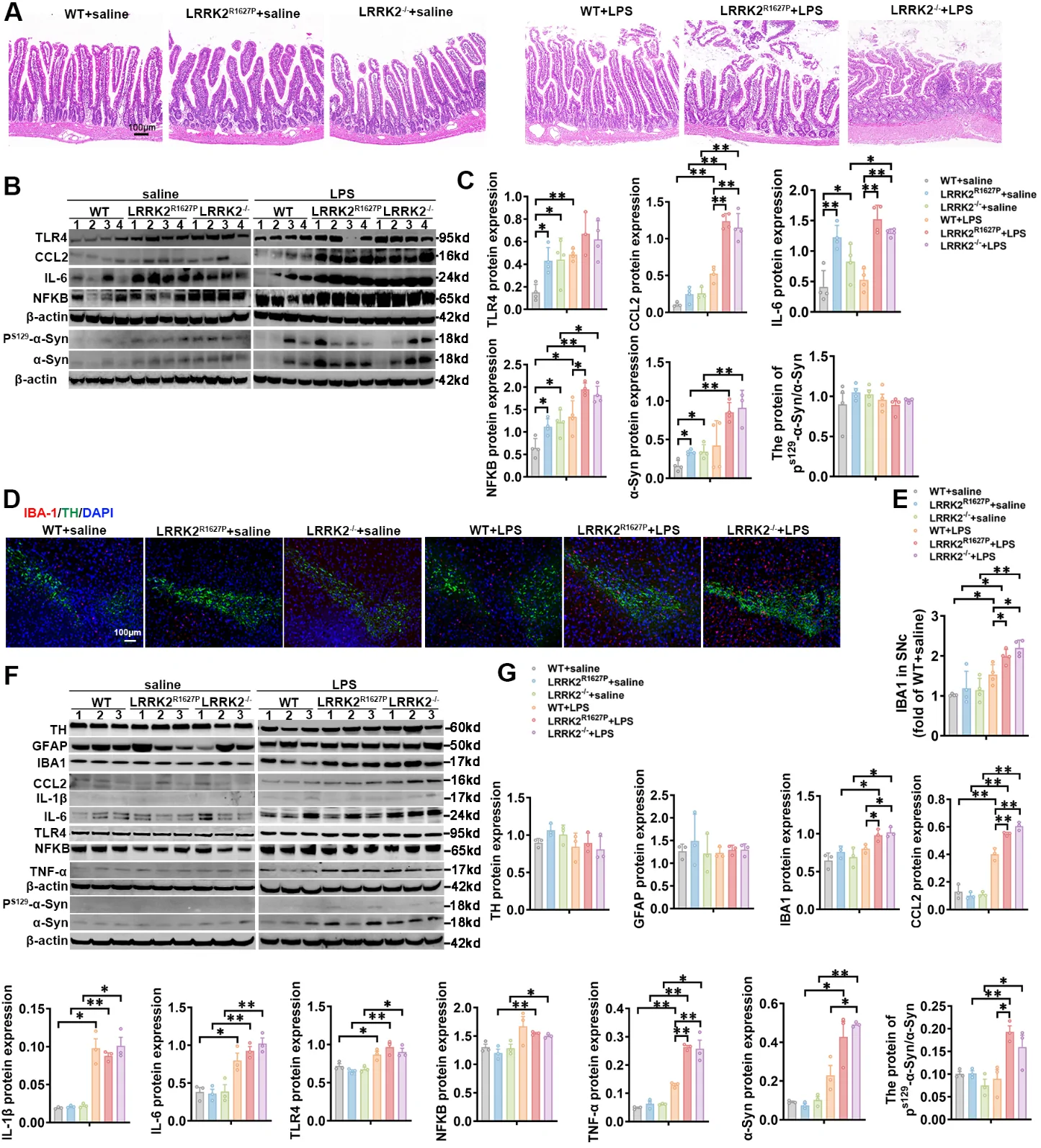
**LPS exacerbates pathological α-Syn aggregation in the small intestine of LRRK2 transgenic rats and spreads to the brain via the gut-brain axis**. (**A**) Representative HE staining images of small intestine in rats treated with saline or LPS. Scale bar 100 μm (N=4). (B, C) Western blot and relative protein expression level of TLR4, CCL2, IL-6, NFKB, p^S129^-α-Syn and α-Syn in the small intestine of rats treated with saline or LPS (N=4 or 5). (**D**) Double immunofluorescence staining images of TH (green) and IBA1 (red) in the SNc of rats treated with saline or LPS. Nuclei were stained with DAPI (blue). Scale bar 100 μm. (**E**) The statistical analysis of IBA1 positive cells in the SNc of rats treated with saline or LPS (N=4). (F, G) Western blot and relative protein expression level of TH, GFAP, IBA1, CCL2, IL-1β, IL-6, TLR4, NFKB, TNF-α, p^S129^-α-Syn and α-Syn in SNc treated with saline or LPS (N=3). Data represent mean ± SD. *P<0.05, **P<0.01.

**Figure 9.**
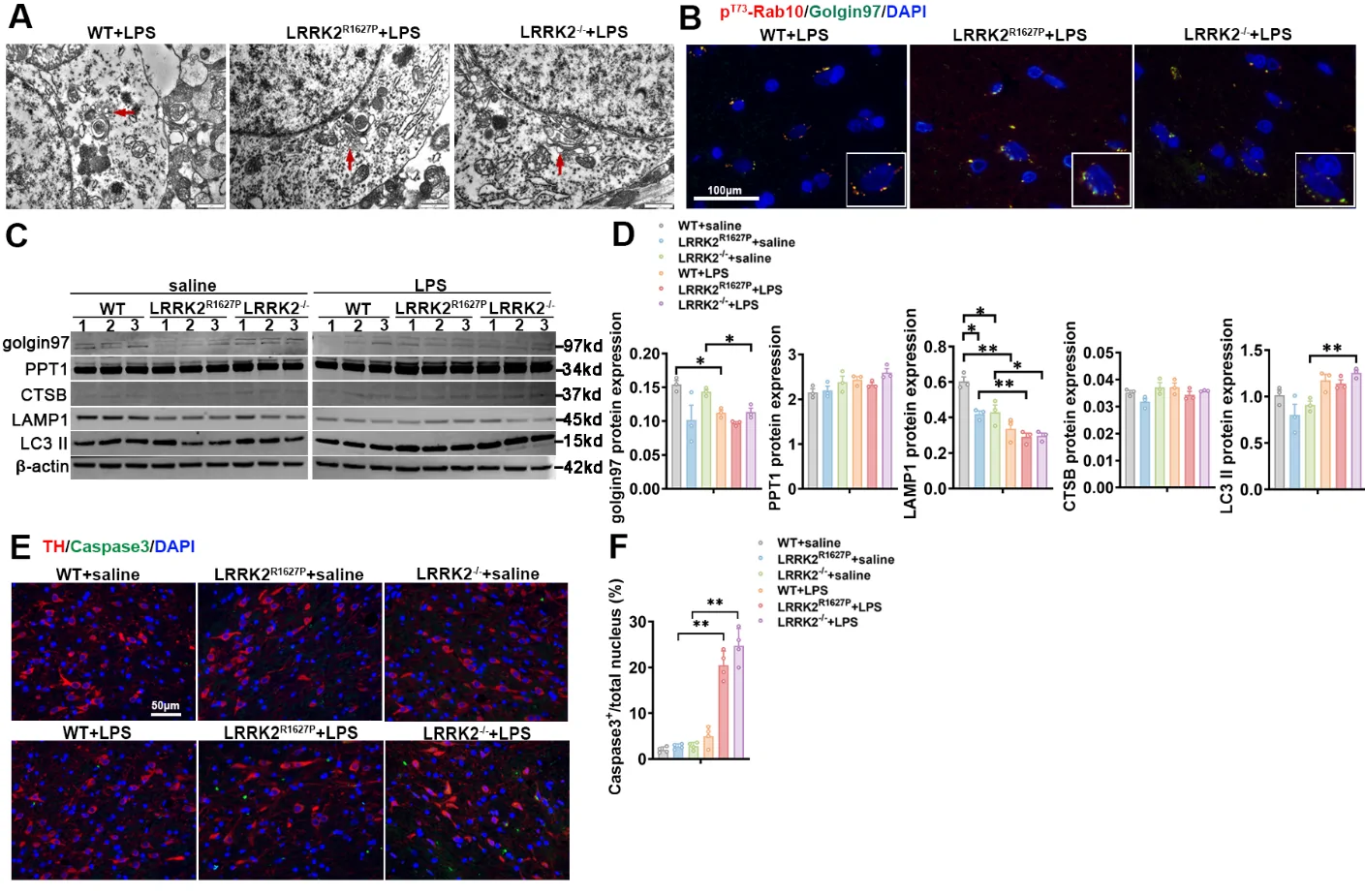
**LPS accelerates cell apoptosis in the brain of LRRK2 transgenic rats via the gut-brain axis**. (**A**) Electron micrographs of the Golgi apparatus and mitochondrial in DANs from SNc after injected LPS (N=3). (**B**) Immunofluorescence of DANs from SNc for Golgin-97 (green) and pT73-Rab10 (red) after injected LPS (N=4). (C, D) WB showed the protein expression of the Golgi apparatus (Golgin-97), lysosomal function (CTSB, PPT1 and LAMP1), and mitochondrial autophagy (LC3-II) from SNc after injected LPS (N=4). (**E**) Immunofluorescence of DANs from SNc for Caspase3 (green) and TH (red) after injected LPS (N=4). (**F**) The statistical analysis of Caspase3 immunofluorescence staining (N=4). Data represent mean ± SD. *P<0.05, **P<0.01.

Considering that our LRRK2 genetic model itself has limited effects on central DANs and motor function in rats, we tried to observe whether rats would develop PD motor symptoms or central pathological α-Syn deposition under the superposition of genes and environmental stimuli. We injected LPS intraperitoneally into rats once a week and observed changes in the intestines and SNc six months later. As shown in Fig. 8A, LPS destroyed the integrity of the small intestinal villi in the three groups of rats, and the intestinal damage in LRRK2 transgenic rats was more severe than that in WT rats. Compared with the control group, western blot results showed that pro-inflammatory mediators (CCL2, IL-6, TLR4, NF-KB) and α-Syn, p^S129^-α-Syn in small intestine of the LRRK2^R1627P^+LPS and LRRK^-/-^+LPS groups were significantly increased (Fig. 8B, C). Although LPS did not cause obvious loss of DANs in rats; however, we found that a significant increase in microglia in the SNc of LRRK2 transgenic rats after injection of LPS (Fig. 8D, E). In addition, compared with the WT+LPS group, LPS did not promote the formation of obvious inflammatory storm in the SNc of LRRK2 transgenic rats, but the expression of pro-inflammatory mediators CCL2, TNF-α, and α-Syn, p^S129^-α-Syn were significantly increased (Fig. 8F, G); and we observed that a large number of mini-stacks were formed in DANs after injection of LPS (Fig. 9A, B). Correspondingly, the expression of Golgin97 and LAMP1 in DANs also decreased (Fig. 9C, D). We further performed TH/Caspase3 immunofluorescence labeling of SNc, and the results showed that apoptotic cells increased significantly in the LRRK2^R1627P^+LPS and LRRK^-/-^+LPS groups (Fig. 9E, F).

## DISCUSSION

**Figure 10.**
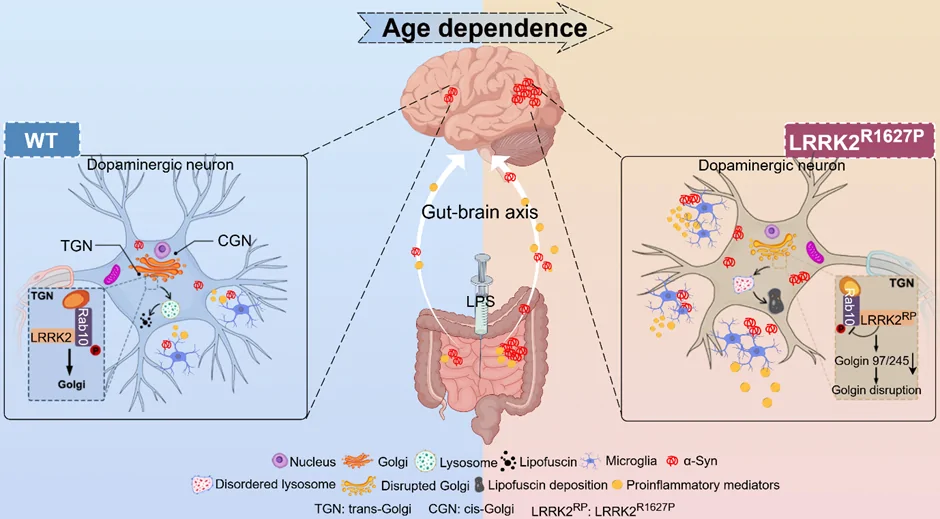
**LRRK2^R1627P^ mutation in rats accelerates dopaminergic neuronal senescence in an age-dependent manner**. The mechanism involves the R1627P mutation disrupting the Golgi structure by inhibiting Rab10 phosphorylation, which in turn impairs the Golgi-lysosome signaling pathway in dopaminergic neurons, accelerating cellular senescence. Moreover, genetic factor (LRRK2^R1627P^ mutation) is more likely to exacerbate intestinal inflammation and pathological α-synuclein aggregation in the rat, when combined with environmental factor (LPS exposure). This pathological phenomenon can spread through the gut-brain axis, leading to microglial activation and the expression of pathological α-synuclein in the brain.

The current studies demonstrate that the rat R1627P variant represents a "loss-of-function" mutation (associated with reduced kinase activity), as evidenced by decreased expression levels of total LRRK2 protein and phosphorylated Rab10. Although LRRK2^R1627P^ rats did not exhibit significant DANs loss, dopamine depletion, or severe motor impairments, they showed diminished spontaneous exploration ability and olfactory discrimination (Table 1). We observed age-dependent dendritic spine degeneration in DANs. Mechanistically, we found that the R1627P mutation in rat inhibited Rab10 phosphorylation on the *trans*-Golgi and disrupting Golgi structure, which played a critical role in the disruption of the Golgi-lysosome signaling pathway, leading to lipofuscin accumulation and accelerating cellular senescence in DANs. Furthermore, although neuroinflammatory features were not significant in the brain of LRRK2^R1627P^ rats, peripheral and intestinal results showed an increase in inflammatory factors, suggesting immune imbalance. This caused abnormal accumulation of α-Syn in the intestines of rats. LPS exacerbates pathological α-Syn aggregation in the small intestine of LRRK2 transgenic rats and spreads to the brain via the gut-brain axis. This leads to an increase in activated microglia in the substantia nigra, creating a pro-inflammatory environment and inducing DANs degeneration (Fig. 10).

**Table 1:** A summary of the neuropathology and behavior of LRRK2 transgenic rats.

Feature	Specification	R1627p	Knockout
	Birth rate	n.s	n.s
	Mortality rate	↑	↑↑ sex (F)
	Number of DAN in SNc	n.s	n.s
	Soma area of DAN in SNc	↓ (24-26m)	↓ ↓ (24-26m)
	Dendrite spines	↓ (24-26m)	↓ ↓ (24-26m)
**Morphology**	Apical dendrite diameter	↓ (8m)	↓ ↓ (8m)
	Lewy body formation	n.s	n.s
	Tau-pathology	n.s	n.s
	Synaptic transmission	n.s	n.s
	Open field	↓ (16m)	↓ (16m)
**Motor symptoms**	Rotarod performance	n.s	n.s
	Grip strength	n.s	n.s
	Anxiety: Forced swim	n.s	n.s
	Object recognition	↓ sex (M) (16m)	↓ sex (M) (16m)
**Prodromal symptoms**	Memory: Morris	↓ sex (F) (16m)	↓ sex (F) (16m)
	Olfactory function	↓ (24-26m)	↓ (24-26m)
	Constipation/diarrhea	n.s	n.s

### Generation of the LRRK2^R1627P^ rat line

Here, our findings reveal that the R1627P mutation has no impact on the mRNA levels of endogenous LRRK2 in rats. However, the protein levels of LRRK2 are notably diminished compared to those in WT rats. This observation implies the possibility of post-transcriptional modifications occurring in LRRK2 mRNA due to the R1627P mutation, which could involve processes such as splicing or RNA editing. Furthermore, it is conceivable that the mutation could lead to reduced translation efficiency by potentially altering the promoter sequence or the translation initiation codon on the mRNA, thus impacting translation initiation efficiency. Another plausible explanation is a decrease in protein stability caused by the R1627P mutation. In contrast to findings in LRRK2^R1627P^ rats, the R1628P mutation did not alter expression of LRRK2 itself in HEK293 cells [6, 7]; However, this does not rule out the presence of other post-transcriptional or post-translational regulatory mechanisms. Exploring potential changes in mRNA splicing, stability, or protein degradation pathways may provide insights into why this mutation manifests a specific effect on LRRK2 levels in vivo rather than in vitro. Moreover, the cellular environment in HEK293 cells may differ from that in tissues, potentially leading to distinct regulatory mechanisms. Conducting additional experiments in primary cells can help reconcile these disparities and offer a more comprehensive understanding of the mechanisms underlying the impact of this mutation on LRRK2. Previous research and our findings confirm that both in transgenic rats and cell models, the phosphorylation levels of the Ser935 residue in LRRK2 activity remain unaffected. Furthermore, due to the lack of specific antibodies for rat-specific autophosphorylation at Ser1292, we were unable to perform detection in this study.

In a recent study, the activity of the R1628P variant located in the CORA domain was analyzed separately from other activating variants, and it was found to stimulate Rab10 phosphorylation by approximately 2-folds [7]. Our results indicate diminished Rab10 phosphorylation in LRRK2^R1627P^ rats’ Str and SNc regions (Fig. 2), suggesting that factors beyond Ser935 phosphorylation might contribute to the regulation of LRRK2 activity in response to the R1627P mutation. These findings also underscore the existence of intricate interactions between LRRK2 expression and activity regulation, such as potential compensatory mechanisms or crosstalk with other signaling pathways that may still mediate LRRK2 activity. Therefore, investigating other phosphorylation sites on LRRK2 and potential crosstalk with signaling pathways could provide a more comprehensive understanding of LRRK2 activity regulation. Compared to previous transgenic mouse models of LRRK2 mutations, such as G2019S [31, 32] and R1441C [31] mutant mice exhibiting an age-dependent decline in Ser935 phospho-levels. Interestingly, R1441C but not G2019S results in increased phospho-Rab10 [10, 33]. Recent studies have suggested that R1441C mutants may be constitutively recruited to Rab10-positive membrane domains independently of Rab29 [31]. Therefore, the reduced Ser935 phosphorylation in LRRK2^R1441C^ mice still ensures the phosphorylation of Rab10. Our study further emphasizes that LRRK2 mutations located in different domains may exert their effects through distinct pathogenic mechanisms. The advantage of using the LRRK2^R1627P^ rat model lies in the maintenance of endogenous phosphorylation levels of LRRK2 protein since birth, which allows exploration of the protein variants and its functional alterations in the course of disease progression over time.

Overall, in this study, our generated R1627P mutant rats showed a significant decrease in Rab10 phosphorylation. This finding is consistent with the notion that the R1628P mutation is considered a 'loss-of-function' rather than a 'gain-of-function'. In contrast, R1441C/R144G mutant mice showed a significant increase in Rab10 phosphorylation, while the G2019S mutation did not affect Rab10 phosphorylation levels [31]. These results suggest that the expression and kinase function enhancement of LRRK2 are controlled by mutations in different domains. This indicates that the pathogenesis of PD may be closely associated with specific LRRK2 functions directly related to the GTPase and kinase domains.

### Pathological analysis

The motor symptoms associated with PD are caused by the loss of DANs in SNc. Consistent with rats overexpressing human LRRK2 mutants, R1627P mutation had no apparent abnormal phenotype, or abnormal parameters in dopaminergic function and protein aggregation even in aged rats (Fig. 3, Supplementary Fig. 2). Further PD associated pathologies such as the formation of LBs, Lewy neurites, up-regulation of α-Syn and neurofibrillary tangles or Tau-pathology in LRRK2^R1627P^ young and old rats were also not detectable in vivo (Fig. 3). Although several studies have reported that LRRK2 mutations or knockout are not accompanied by loss of DANs, they have consistently observed alterations in the morphology or function of these neurons. Such as, G2019S mutation induces dendritic shortening and DANs death [34, 35]. The DANs in R1441C mice do not show morphological alterations at the postsynaptic side of the nigrostriatal pathway, whereas LRRK2 knockdown neurons exhibit subtle trends towards an increase in branching and network complexity [28]. It is intriguing to note that, while the DANs count was similar across various rat genotypes (Fig. 3), there was an age-dependent reduction in both dendritic spine density and size within the DANs of both LRRK2^R1627P^ and LRRK2^-/-^ rats (Fig. 4). These observations are in line with the findings reported by Loukia Parisiadou [36] and Chen [37] et al. These results confirm that the R1627P mutation in rats or endogenous LRRK2 deficiency leads to a decrease in the number of mature spines and alterations in synaptic transmission. The diverse spectrum of pathogenic mutations within the multiple domains of LRRK2 protein and the complex mechanisms by which these mutations influence the development of PD, including changes in LRRK2 kinase activity, alterations in autophagic activity, and modifications in the clearance capacity of α-Syn protein, etc. In summary, whether it is LRRK2 'gain-of-function' or 'loss-of-function', these changes may lead to an imbalance in protein homeostasis within neuronal cells, resulting in varying degrees of neuronal damage. Therefore, investigating the molecular mechanisms underlying neuronal degeneration caused by different structural domain mutations of LRRK2 can contribute to a deeper understanding of the impact of LRRK2 on neuronal health and provide a theoretical basis for the development of related therapeutic strategies.

Notably, the high mortality rate observed in

LRRK2^R1627P^ and LRRK2^-/-^ female rats at 22-month precluded further comprehensive examination of the aging-related effects on the brain in aged rats with LRRK2 loss. Dissection examination revealed that the high mortality rate in LRRK2^R1627P^ and LRRK2^-/-^ female rats may be associated with tumors (data not shown). Consistent with previous studies, LRRK2^R1627P^ and LRRK2^-/-^ rats exhibited age-dependent renal atrophy [38, 39]. At 2-month, the kidneys of LRRK2^R1627P^ and LRRK2^-/-^ rats showed a darker color, splenomegaly, and muscle atrophy (Supplementary Fig. 10). These findings indicate that reduced or absent endogenous LRRK2 leads to damage in peripheral tissues.

### Behavioral analysis

The open field and swimming tests can assess spontaneous exploration, motor coordination, and emotional responses following exposure to a novel environment in rodents [40]. Other psychiatric symptoms, which affect approximately 40% of PD patients, may precede the onset of motor symptoms [41]. Although LRRK2 mutations are considered a key genetic risk factors for PD, studies have shown that some LRRK2 mutations or knockout mice/rats do not exhibit motor symptoms, as summarized by Steven P. Seegobin et al. [42]. The aforementioned pathological findings indicate that despite an age-dependent reduction in dendritic spine quantity within substantia nigra neurons of LRRK2^R1627P^ and LRRK2^-/-^ rats, no apparent abnormalities were observed in dopamine and its metabolites (Supplementary Fig. 3, 4). The open field test found that the moving distance and speed of these two strains of rats decreased in an age-dependent, and their trajectories in exploring the center of the open field were significantly less than those of the WT (Fig. 6 and Supplementary Fig. 6), whereas there were no significant differences observed in the swimming experiment (Supplementary Fig. 7). Combining pathological and behavioral analyses, our results indicate that while the reduction in dendritic spine density in DANs may affect synaptic transmission and lead to behavioral changes in both strains of rats, it does not necessarily imply an impact on dopamine levels, as neurons are still capable of releasing sufficient dopamine to maintain function. Given that several LRRK2 mutation models exhibit normal total dopamine levels but subtle defects in extracellular release [43-45], this prompts us to investigate future studies examining the extracellular release of dopamine and other neurotransmitters in LRRK2^R1627P^ and LRRK2^-/-^ rats, which may provide further insights into the abnormal behavior observed in transgenic rats in the open field. It is also important to note that the impact of LRRK2 on rat behavior is likely to be complex and influenced by various factors such as age, gender, environment, and different genetic backgrounds of strains. Therefore, behavioral differences may arise in LRRK2 mutant or knockout transgenic mice/rats.

### Regulation of the Golgi-lysosome pathway by the R1627P mutation in rats

Previous studies have established the role of LRRK2 in several key intracellular processes, including Golgi integrity, vesicle trafficking, vesicle endocytosis, and autophagy [9, 11, 12, 46, 47]. Our study provides further insight into the importance of the LRRK2 protein in regulating intracellular Golgi and lysosomal function, and its impact in relation to the R1627P mutation in rats. Our findings are consistent with these previous findings, emphasizing the importance of LRRK2 in maintaining the normal function of organelles. Studies have also found that inactivation of LRRK2 not only leads to fragmentation of the Golgi apparatus and disturbance of vesicle transport [11], but may also disrupt the function of the entire endolysosomal system, including transport from the Golgi apparatus to lysosomes and endocytosis [12]. Golgi apparatus is an important part of the internal system of the cell, and its dysfunction may have a wide range of effects on the function of endosomes and lysosomes, and the transport of synaptic vesicles, thereby changing the function of neurons and synaptic plasticity [12]. In particular, our study of R1627P mutant rats revealed that this mutation disrupts the structure of the Golgi apparatus by inhibiting the phosphorylation of Rab10, resulting in the loss of Golgi apparatus strips and the formation of "minimal stacks", as well as the aberrant accumulation of vesicles in trans-Golgi region in neurons (Fig. 7). Rab10 localizes in the Golgi apparatus and early endosomes and functions in retrograde Golgi-to-plasma membrane transport and phagosome maturation [48, 49]. Its phosphorylation status can affect its cellular localization and function [30, 50]. Through quantitative analysis, we found that Golgi-associated proteins, such as CTSB, PPT1, and LAMP1, were down-regulated (Fig. 7). These proteins play key roles in maintaining homeostasis in cells, protein degradation, and cell metabolism. Our study further supports the critical role of Rab10 in cells, pointing out that the reduced phosphorylation of Rab10 triggered by the R1627P mutation may interfere with the communication and fusion between the Golgi apparatus and lysosomes, resulting in the accumulation and aggregation of vesicles, impacting cellular metabolism and clearance functions, which may be early contributors to the PD pathogenesis. This abnormality may be particularly acute in neurons, where Rab10 also regulates intracellular vesicle trafficking, mediates axonogenesis [51] and dendrite branching [52, 53]. This can lead to abnormal accumulation of intracellular material and deposition of lipofuscin, which eventually leads to atrophy and degeneration of neuronal dendrites. Furthermore, we note that some studies have shown that Rab10 hyperphosphorylation may be associated with the development of neurodegenerative diseases in some cases [30]. Therefore, maintaining the normal phosphorylation status of Rab10 may be of potential benefit in the prevention or treatment of neurodegenerative diseases associated with dysfunction of the Golgi-lysosome pathway. This also means that modulating the phosphorylation status of Rab10 through drugs or therapeutic strategies may become a new way to treat these diseases. Our study provides new insights into the roles of LRRK2 and Rab10 in the nervous system, as well as the pathological mechanisms of related diseases. Although we have made important progress in this study, further studies are still needed to reveal more detailed molecular mechanisms and the possibility of how to apply these findings to treatments.

### The R1627P mutation in rats serve as a key link between the peripheral inflammation-gut-brain axis (PI-GBA) and neurodegenerative diseases

The association between the gastrointestinal system and the pathogenesis and progression of PD has become increasingly recognized. It has been proposed that various types of gastrointestinal disturbances leading to intestinal inflammation may promote peripheral inflammation, subsequently contributing to neuroinflammation and neuropathology associated with PD. Mutations in LRRK2 have been implicated in the increased incidence of both PD and Crohn's disease (CD). Multiple lines of evidence indicate that LRRK2 is also highly expressed in immune cells [54, 55]. Based on the observation of elevated levels of LRRK2 in inflamed colonic tissues of patients with CD and in peripheral immune cells of individuals with idiopathic PD compared to controls, some studies suggest that the higher levels of LRRK2 in intestinal inflammation and CD could serve as biomarkers for increased risk of idiopathic PD [56, 57]. In the subgroup of asymptomatic individuals carrying the G2019S mutation, higher levels of peripheral pro-inflammatory cytokines have been observed [58]. Taken together with the finding of increased overall LRRK2 levels in immune cells of patients with idiopathic PD [54], it suggests that LRRK2-driven inflammation plays an early role in PD. Our study indicates that the immune system alterations, particularly the decreased ratio of CD4^+^/CD8^+^ T cells and B cells in LRRK2^R1627P^ rats, might contribute to the neuroinflammatory response in PD. This peripheral immune imbalance, combined with the central inflammation, creates a systemic environment that may be detrimental to neuronal survival and function [59]. In addition, the LRRK2^R1627P^ mutation or knockout causes autoimmune diseases and malignant tumors, suggesting that LRRK2^R1627P^ and LRRK2^-/-^ rats have higher mortality due to tumors. These findings are consistent with the results from Shutinoski MH et al. [60], which showed limited bacterial burden and increased mortality in LRRK2 knockout mice. Immunofluorescence labeling showed increased infiltration of M1 macrophages and elevated levels of inflammatory factors in the the small intestine of 16-month LRRK2^R1627P^ and LRRK2^-/-^ rats (data not shown). In both strains of rats, peripheral inflammatory features preceded intestinal. These findings suggest that the R1627P mutation in rats or LRRK2 deficiency can induce peripheral inflammation, which may promote intestinal inflammatory infiltration, influence the composition and function of the gut microbiota, and increase intestinal permeability. We speculate that these changes may lead to the entry of harmful substances (such as endotoxins) and bacteria from the intestine into the circulatory system, potentially breaching the BBB and affecting the function of the central nervous system. However, in this study, no signs of neuroinflammation or neuronal loss were observed in the brains of LRRK2^R1627P^ and LRRK2^-/-^ rats under physiological conditions (Supplementary Fig. 5). On one hand, this may be attributed to the relatively short lifespan of rats compared to humans, particularly in neurodegenerative diseases like PD, where disease symptoms progress over time. On the other hand, it implies that LRRK2 mutations causing peripheral inflammation, intestinal inflammation, and disorder of intestinal flora may be crucial factors in breaching the BBB and contributing to the progression of PD. The R1627P mutation, in concert with LPS-induced inflammation, exacerbates α-Syn pathology in the gut and brain. This supports the theory by Liang et al that environmental factors can potentiate genetic predispositions to LRRK2 risk variants [61, 62]. The exacerbated inflammation and α-Syn accumulation in LRRK2^R1627P^ rats' intestines and the subsequent spread to the brain via the gut-brain axis underscore the role of peripheral inflammation in central neurodegenerative processes [63]. Moreover, the phenomenon of microgliosis and elevated apoptotic cells in the SNc of LRRK2^R1627P^ rats following LPS treatment points towards an inflammatory environment conducive to neurodegeneration. Therefore, understanding how the LRRK2^R1627P^ mutation in rats break through the BBB is crucial in establishing the connection between PI-GBA. It can also serve as a basis for determining whether clinical LRRK2^R1628P^ mutation patients develop and contribute to neurodegeneration in PD. Overall, these data highlight the significant role of LRRK2 in the PI-GBA, emphasizing the need for future research to explore how LRRK2 interacts with intestinal inflammation to promote neuroinflammation and neuropathology associated with PD.

### Conclusion

In summary, we have established a transgenic rat model expressing the R1628P mutation of human LRRK2 and elucidated the role of the LRRK2 COR domain in vivo. Unlike functionally 'gain-of-function' mutant LRRK2 transgenic mice that increase molecular mechanisms triggering cytotoxicity, our results reveal that the R1627P mutation in rats perturbs Golgi structure by inhibiting Rab10 phosphorylation, thereby disrupting the Golgi-lysosome signaling pathway in DANs and accelerating cellular senescence. The study uniquely contributes to the understanding of PD by showcasing the mutation's peripheral effects. Particularly, we highlight an age-dependent pro-inflammatory environment in the gut, accentuated by environmental stimuli such as LPS, which exacerbates the pathological accumulation of α-Syn. The subsequent spread of this pathology to the brain via the gut-brain axis emphasizes the significance of peripheral inflammation in central neurodegenerative processes. Our findings strongly advocate for the need to monitor gut-brain axis integrity, especially in individuals harboring LRRK2 mutations. The results demonstrate that while the LRRK2^R1627P^ mutation itself has limited effects on central DANs and motor functions, its interaction with environmental factors can lead to significant pathological changes. These changes are instrumental in unraveling the intricate relationship between genetic predispositions and environmental influences in PD pathogenesis. Our investigations underscore the clinical implications of the LRRK2 mutation, laying a foundation for future research aimed at developing targeted therapeutic interventions. Addressing the gut-brain axis and its integrity may hold the key to innovative treatment strategies for PD, potentially slowing or preventing disease progression in at-risk populations. The LRRK2^R1627P^ rat model serves as a valuable tool for such endeavors, providing insights into the early stages of PD and potential intervention points to hinder its advancement.

## Supplementary Materials

The Supplementary data can be found online at: www.aginganddisease.org/EN/10.14336/AD.2024.0883.

## Data Availability

Data will be made available on request.
