# Supplementary material for: Impairment of the trans-Golgi-Lysosomal Pathway Accelerates Dopaminergic Neuronal Senescence in LRRK2R1627P Rats
Source: Aging Dis. 2024 Oct 27;16(5):3089–111. doi: 10.14336/AD.2024.0883 (PMC12339174; doi:10.14336/AD.2024.0883)
Supplement: Supplementary file 1 — The Supplementary data can be found online at: www.aginganddisease.org/EN/10.14336/AD.2024.0883. [file AD-16-5-3089-s.pdf]

SUPPLEMENTARY DATA

Impairment of the *trans*-Golgi-Lysosomal Pathway Accelerates Dopaminergic Neuronal Senescence in LRRK2^{R1627P} Rats

Qiumei Yang, Shimin Pang, Chunsong Zhao, Yanyan Wang, Jing Lu, Zhenyu Yue, Piu Chan

© 2024. Yang Q et al. Published online at http://www.aginganddisease.org/EN/10.14336/AD.2024.0883

SUPPLEMENTARY DATA

Materials and Methods

Reagents: Both protease and phosphatase inhibitors were purchased from Roche (Basel, Switzerland, Cat#4693132001 and Cat#04906837001). Primary antibodies used in western blotting are as follows: Rabbit anti LRRK2 (1:500, Abcam, Cat#ab133474), Rabbit anti p^{Ser935}-LRRK2 (1:500, ABclonal, Cat#AP1154), Rabbit anti Rab10 (1:500, Abcam, Cat#ab237703), Rabbit anti p^{T73}-Rab10 (1:500, Abcam, Cat#ab241060), Mouse anti Rab8a (1:200, Santa Cruz, Cat#sc-81909), Rabbit anti p^{T72}-Rab8a (1:500, Abcam, Cat#ab230260), Rabbit anti Rab5a (1:500, affinity, Cat#DF6314), Mouse anti Rab12 (1:200, Santa Cruz, Cat#sc-515613), Rabbit anti DAT (1:500, Proteintech, Cat#22524-1-AP), Rabbit anti VAMP2 (1:500, GeneTex, Cat#GTX133241), Rabbit anti α-Syn (1:500, CST, Cat#4179), Rabbit anti p^{S129}-α-Syn (1:500, CST, Cat#23706), Mouse anti TH (1:500, Invitrogen, Cat #MA1-2465), Mouse anti Drebin (1:200, Santa Cruz, Cat#sc-374269), Mouse anti MAP2 (1:250, Invitrogen, Cat #MA1-2504), Rabbit anti MAP1B(1:500, GeneTex, Cat#GTX116014), Rabbit anti NFKB (1:500, affinity, Cat#AF6217), Rabbit anti TNF-a (1:500, affinity, Cat#AF7014), Mouse anti INF-r (1:200, Santa Cruz, Cat#sc-12755), Rabbit anti TLR4 (1:500, Servicebio, Cat#GB11519), Rabbit anti TGF-beta (1:500, affinity, Cat#AF1027), Rabbit anti CXCL1 (1:500, Proteintech), Mouse anti golgin-97 (1:200, Santa Cruz, Cat#sc-59820), Rabbit anti golgin-84 (1:500, Servicebio, Cat#GB112721), Mouse anti golgin-160 (1:200, Santa Cruz,Cat#sc-374596), Mouse anti golgin-245(1:1000, CST,Cat#79145), Rabbit anti GCC-88 (1:500, Boster,Cat#A12100-1), Mouse anti Cathepsin B (CTSB) (1:200, Santa Cruz,Cat#sc-365558), Rabbit anti PPT1 (1:500, Invitrogen,Cat#PA5-29177), Rabbit anti LAMP1 (1:500, affinity, Cat#DF4806), VDAC1(1:1000, Proteintech,Cat#55259-1-AP), Rabbit anti LC-II (1:500, CST, Cat#2775), Rabbit anti synaptophysin (SYP) (1:1000, Servicebio, Cat#GB12814), Rabbit anti D2R (1:300, Invitrogen, Cat#55084-1-AP), Mouse anti-β-actin (1:1000, Servicebio, Cat#GB12001), Rabbit anti-β-actin (1:1000, Servicebio, Cat#GB11001), Mouse anti-GAPDH (1:1000, Servicebio, Cat#GB12002), Rabbit anti-GAPDH (1:2000, Servicebio, Cat#GB11002).

The following antibodies were used for immunocyto/histochemistry: Rabbit anti α-Syn (1:200, CST, Cat#4179), Rabbit anti p^{S129}-α-Syn (1:200, CST, Cat#23706), Mouse anti TH (1:200, Invitrogen, Cat #MA1-2465), Mouse anti IBA-1 (1:200, Servicebio, Cat#GB15105), Rabbit anti p^{T73}-Rab10 (1:200, Abcam, Cat#ab241060), Mouse anti golgin-97 (1:100, Santa Cruz, Cat#sc-59820), Rabbit anti GFAP (1:200, Servicebio, Cat#GB11096).

The following antibodies were used for fluorescence-activated cell sorting (FACS): CD11b-FITC (Biolegend, Cat#554982), CD68-APC (Novus, Cat#NB100-683APC), CD163-PerCP (Novus, Cat#NBP2-39099PCP), CD3-FITC (Biolegend, Cat#201403), CD4-PerCP (Biolegend, Cat#201520), CD8-APC (Novus, Cat#NBP2-12523APC), and CD22-Alexa Fluor®647 (Novus, Cat#FAB2296R).

Secondary antibodies are as follows: Goat anti-Mouse 680 Alexa Fluor Plus (1:50000, Thermo Fisher Scientific, Cat#A28183), Goat anti-Rabbit 800 Alexa Fluor Plus (1:50000, Thermo Fisher Scientific, Cat#A32735), Goat anti-Rabbit 680 Alexa Fluor Plus (1:50000, Thermo Fisher Scientific, Cat#A32734), Goat anti-Mouse 800 Alexa Fluor Plus (1:50000, Thermo Fisher Scientific, Cat#A32730), AlexaFluor 488-conjugated goat anti-mouse IgG (1:1500, Thermo Fisher Scientific, Cat#A28175), AlexaFluor 488-conjugated goat anti-rabbit IgG (1:1500, Thermo Fisher Scientific, Cat#A-1108), AlexaFluor 647-conjugated goat anti-mouse IgG (1:1500, Thermo Fisher Scientific, Cat#A-21235), AlexaFluor 647-conjugated goat anti-Rabbit (1:1500, Thermo Fisher Scientific, Cat#A-21244).

Generation of LRRK2^{R1627P} knock-in and LRRK2$^{-/-}$ rat: LRRK2^{R1627P} knock-in rats were generated by Biocytogen, Inc. using EGE(CRISPR/Cas9) system methodology. In brief, to generate the gene targeting vector, single guide RNAs (sgRNAs) were designed in the regions of intron33-34 and intron34-35 of the LRRK2 gene, respectively. For each targeting site, candidate sgRNAs were designed by the CRISPR design tool (http://crispr.mit.edu). The nucleic acid change, c.4880G>C, was introduced into the endogenous LRRK2 rat locus using sgRNA (5'-AGAATGAATGCCGGAGATAGGGG-3') and (5'-AGAGAATCCTCATATTGCTCAGG-3'), and the repair oligonucleotide (5'-CTATTTCTAGCTCTAAAACCTATCTCCGGCATTCATTCCGGT GTTTCGTCCTTTCCA-3') or (5'-CTATTTCTAGCTCTAAAACGAGCAATAT GAGGATTCTCCG GTGTTTCGTCCTTTCCA-3'), which mutates the rat amino acid position 1627 Arg to Pro (CGG to CCG). Candidate sgRNAs were screened for on-target activity using UCA™ (Universal CRISPR Activity Assay), a

© 2024. Yang Q et al. Published online at http://www.aginganddisease.org/EN/10.14336/AD.2024.0883

sgRNA activity detection system developed by Biocytogen. The Cas9 mRNA and sgRNAs were transcribed by T7 RNA polymerase by PCR amplification, gel purified, and used as the template for in vitro transcription using the MEGAshortscript T7 kit (Life Technologies) according to the kit protocol. To minimize random integration, PCR and Southern blot were used to verify gene-targeted rats with correct recombination, and F1 generation positive rats were verified using both 5' Probe-D (Fwd: 5'-CCACTGGGTTTCACTCATTTGTGGC-3', Rev: 5'-TGCCATCTGTAGCATTTGGGCAGAA-3') and RR Probe-A (Fwd: 5'-AGTAGTTATTTCCCTGGG ATTGAGGCG-3', Rev: 5'-ACCTCTTCCAGAAAGCATGAAAGGTGA-3'). RR Probe-A was used to detect random insertions. BglII and NcoI were used as restriction sites for Southern blot. 5' Probe-D was used to detect correct recombination. Optimized concentrations of Cas9 mRNA, sgRNAs, and donor vector were mixed and co-injected into SD rat embryos, which were implanted into pseudo-pregnant females. Germline transmission was verified in a single founder by genotyping and sequencing the F1 pups. Rats were backcrossed to SD rat for four generations to reduce transmission of any off-target mutations, prior to setting up the heterozygous × heterozygous cross to obtain heterozygous, homozygous and WT littermates.

LRRK2$^{-/-}$ knockout rats were obtained from non-homologous end-joining using sgRNA (5'-AGAATGAAT GCCGGAGATAGGGG-3', or 5'-AGAGAATCCTCATATTGCTCAGG-3'. Germline transmission was verified by genotyping and sequencing F1 pups, prior to backcrossing to SD for four generations and intercrossing.

Genotyping of LRRK2R1627P knock-in rat: Genomic DNA was extracted from tail biopsies of rats, and genotype identification by Sanger sequencing of PCR amplification products using extracted genomic DNA. Primers: Fwd: 5'-GAGAGGTTGTTTAAGCTGGGAAGCA-3'; Rev 5'-GAGTGTTAAGCCAAAAGAACA ACTGTATGC-3'.

Genotyping of LRRK2$^{-/-}$ rat: Genotyping was performed on tail biopsy DNA from rats using allelic discrimination PCR assay. Primers: WT: Fwd: 5'-AAGATTAATGGCGAAGCCGTGTGTG-3'; Rev 5'-TGGCATGTGATCGTCTACATCAAGAA-3'; deleted LRRK2 allele: Fwd: 5'-AATGATGCTGAAT TCCCCCTGGCAT-3'; Rev: 5'-TGGCATGTGATCGTCTACATCAAGAA-3'.

RNA extraction and qRT-PCR: Total RNA was extracted from brain using the TRIzol reagent (invitrogen) and reverse-transcribed with reverse transcriptase (Promega). qRT-PCR analyses were performed in triplicate using the LightCycler 480 SYBR Green I qPCR Master Mix (Roche). GAPDH was used as an internal control for qRT-PCR analyses. Primers: LRRK2 Fwd:5'-CGAAGGAGAGGAAGTGGCTG-3'; Rev: 5'-GCTGGG ATGGTGGAGATGAC-3'. GAPDH Fwd:5'-TGGCCTTCCGTGTTCCTACC-3'; Rev: 5'-CGCCTGCTTCA CCACCTTCT-3'.

Western blot analysis: Tissues were homogenized using low temperature tissue grinder at 2100 rpm in RIPA lysis buffer supplemented with Roche cOmplete Protease Inhibitors and PhosSTOP Phosphatase Inhibitors (Roche). The supernatants were normalized to total protein concentration using a BCA protein assay kit (Thermo Fisher Scientific). Proteins were separated by SDS-polyacrylamide gel electrophoresis (SDS-PAGE) and transferred to PVDF membrane. The membranes were blocked with 5% BSA for 2 h at room temperature, and subsequently probed with primary antibodies overnight at 4°C.

Histology, Immunohistochemistry and Immunostaining: Rats were perfused intracardially with 0.9% saline and then 4% paraformaldehyde (PFA) in PBS solution (PFA/PBS). The brains were removed, immersed in 4% paraformaldehyde overnight. Subsequently, the tissue samples were embedded and sliced. Brain sections were subjected to H2O2-inactivation of endogenous peroxidase activity and treated with 5% BSA in PBS-0.2% Triton X-100 for 60 min at room temperature to block non-specific protein binding. For immunohistochemical analysis, the tissue sections were incubated with primary antibodies (diluted in 5% goat serum/0.1% Triton X-100/PBS) for 2h. A biotinylated secondary antibody against rabbit/mouse IgG was used together with an ABC kit (Vector Laboratories) for detection. Optical density was analyzed using NIH ImageJ software. For immunofluorescence staining, the tissue sections were incubated with primary antibodies (diluted in 5% goat serum/0.2% Triton X-100/PBS) overnight at 4°C, and subsequently with fluorescein isothiocyanate (FITC) or phycoerythrin (PE)-conjugated appropriate secondary antibodies for 1h at room temperature.

© 2024. Yang Q et al. Published online at http://www.aginganddisease.org/EN/10.14336/AD.2024.0883

SUPPLEMENTARY DATA

Schematic figure of experimental design

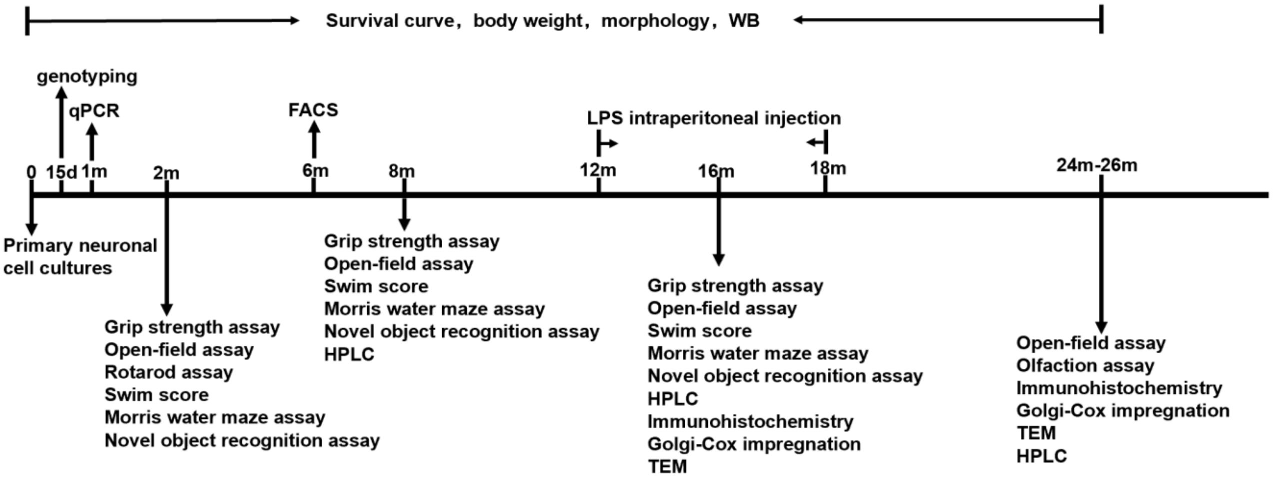

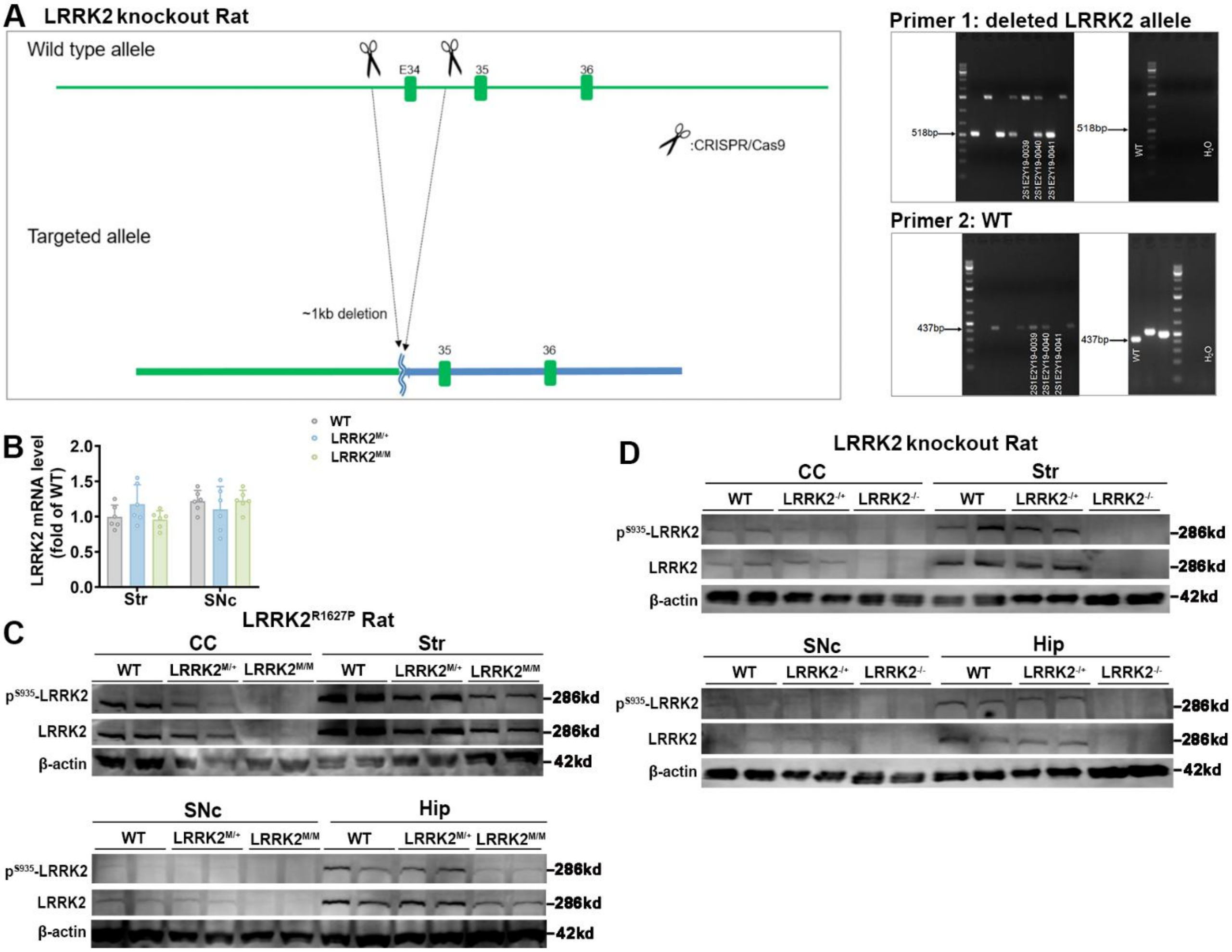

© 2024. Yang Q et al. Published online at http://www.aginganddisease.org/EN/10.14336/AD.2024.0883

SUPPLEMENTARY DATA

Supplementary Figure 1. LRRK2 protein expression level decreased in R1627P knock-in rats. (A) Genomic structure of the rat LRRK2 allele and the gene targeting strategy by CRISPR-Cas9. (B) Real-time PCR shows the expression of LRRK2 in the brain of WT, LRRK2$^{M/+}$ and LRRK2$^{M/M}$ at 3-weeks-old. Six animals of each group were quantified (N=6). (C) LRRK2 protein levels in the brain of WT, LRRK2$^{M/+}$ and LRRK2$^{M/M}$ at 3-weeks-old by WB (N=4). (D) LRRK2 protein levels in the brain of WT, LRRK2$^{-/+}$ and LRRK2$^{-/-}$ at 3 weeks of age by WB (N=4).

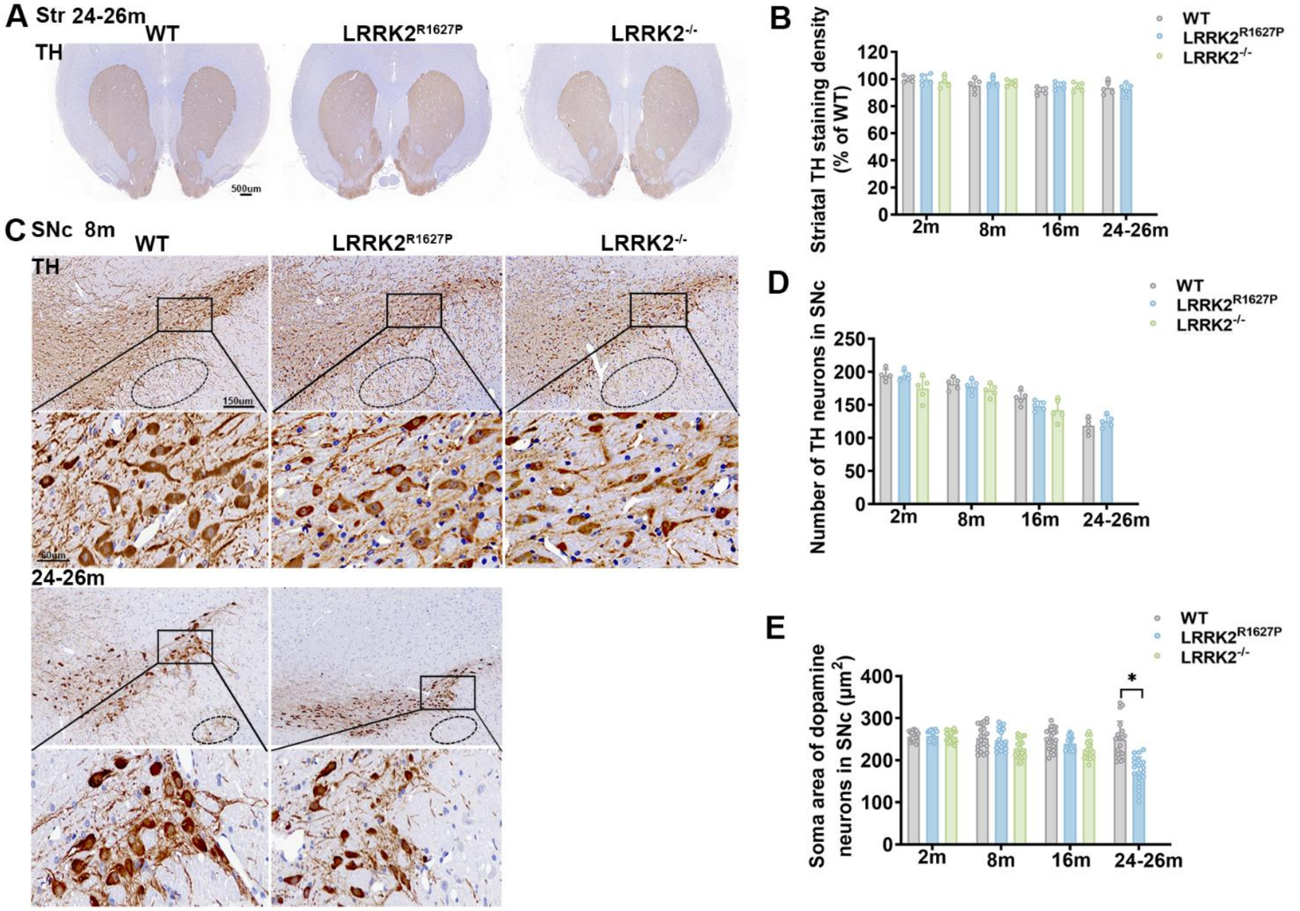

Supplementary Figure 2. Age-dependent reduction in soma area of DA neurons in LRRK2^{R1627P} female rats. (A/C) No apparent difference in the expression pattern of TH in Str and SNc at 24-26-month-old between WT and LRRK2^{R1627P} or LRRK2$^{-/-}$ female rats (N = 5). Scale bar: 500μm in (A) and 150μm in (C) (N=5). (B/D) There were no statistical differences in intensity or number of staining between TH^{+} DA neurons at Str (B) and SNc (D) (N=5). (E) Statistical analysis of soma area of TH^{+} DA neurons in the SNc of different ages (N=5). Data represent mean ± SD. *P<0.05.

© 2024. Yang Q et al. Published online at http://www.aginganddisease.org/EN/10.14336/AD.2024.0883

SUPPLEMENTARY DATA

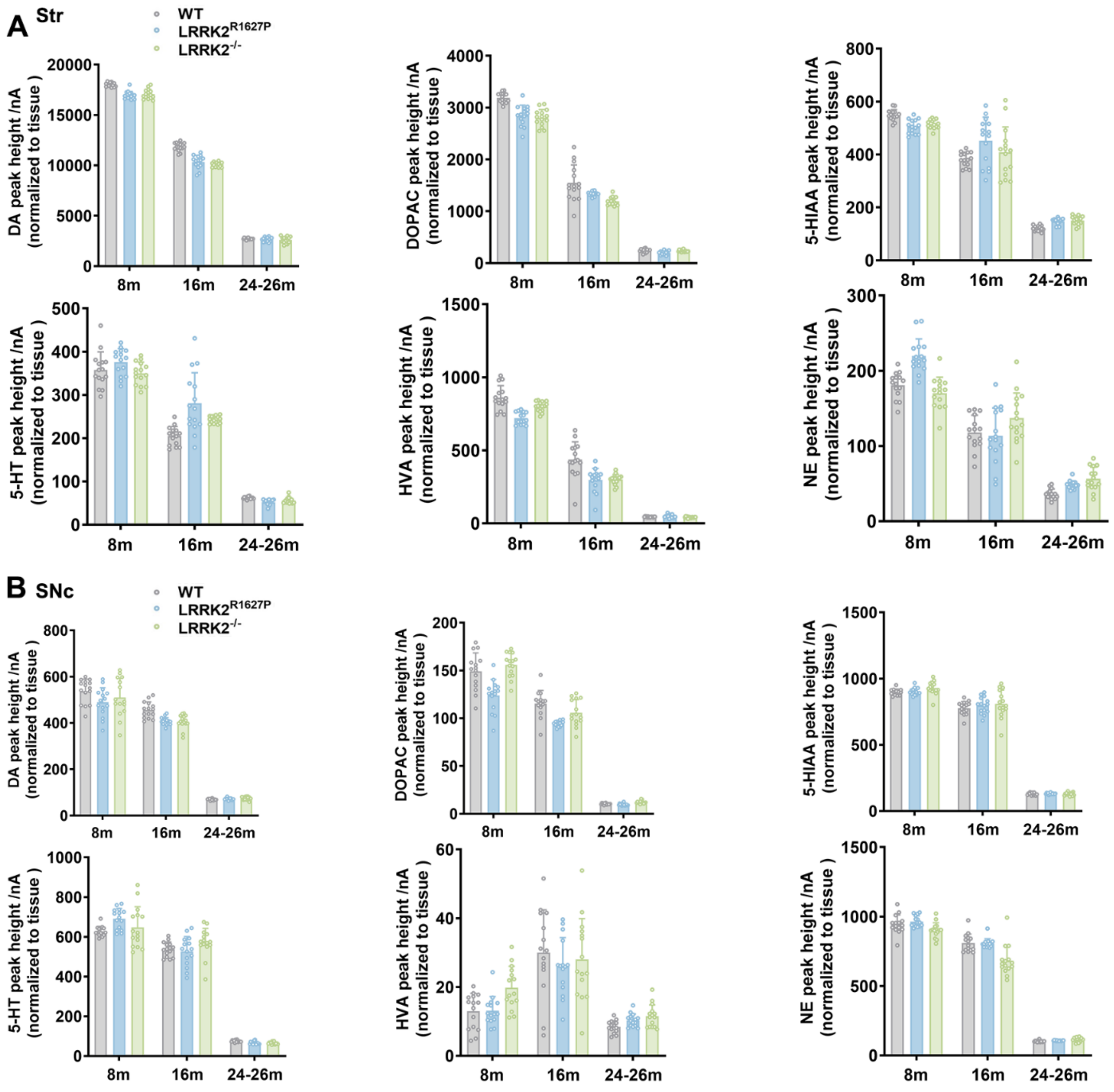

Supplementary Figure 3. Dopaminergic characterization in LRRK2^{R1627P} and LRRK2$^{-/-}$ male rats. Dopamine axon terminal neurochemistry is normal in LRRK2^{R1627P} and LRRK2$^{-/-}$ rat. HPLC analysis of Str (A) and SNc (B) content of total DA and its metabolites DOPAC, 5-HIAA, 5-HT, HVA and NE (N=15). Data represent mean ± SD.

© 2024. Yang Q et al. Published online at http://www.aginganddisease.org/EN/10.14336/AD.2024.0883

SUPPLEMENTARY DATA

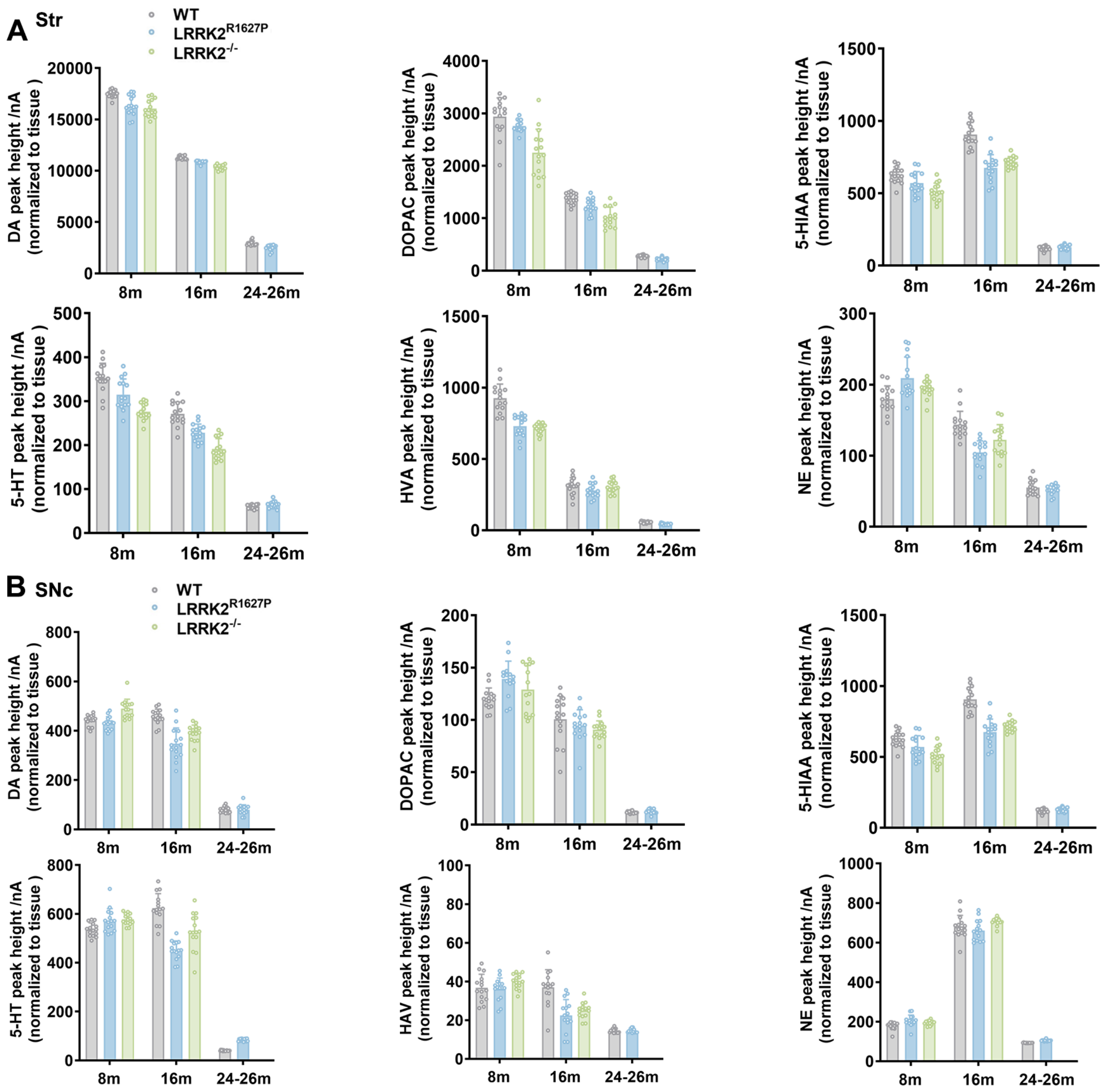

Supplementary Figure 4. Dopaminergic characterization in LRRK2^{R1627P} and LRRK2$^{-/-}$ female rats. Dopamine axon terminal neurochemistry is normal in LRRK2^{R1627P} and LRRK2$^{-/-}$ rat. HPLC analysis of Str (A) and SNc (B) content of total DA and its metabolites DOPAC, 5-HIAA, 5-HT, HVA and NE (N=15). Data represent mean ± SD.

© 2024. Yang Q et al. Published online at http://www.aginganddisease.org/EN/10.14336/AD.2024.0883

SUPPLEMENTARY DATA

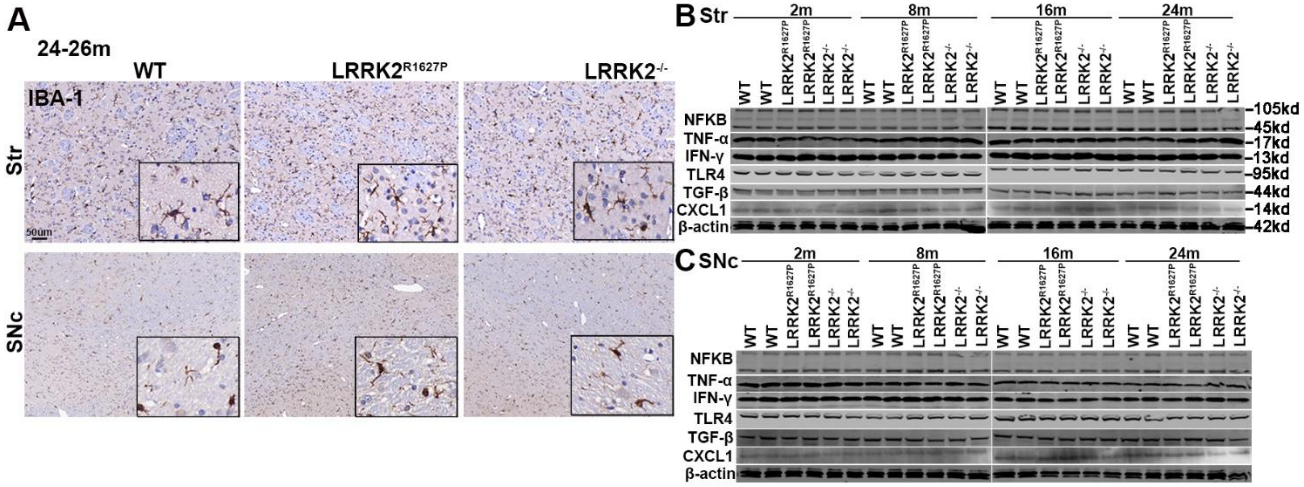

Supplementary Figure 5. No significant changes in the inflammatory features in Str and SNc of LRRK2 transgenic rats. (A) No apparent difference in the expression pattern of IBA-1 in Str and SNc at 24-26-month-old between WT and LRRK2^{R1627P} or LRRK2-/- rats (N = 5). Scale bar: 50μm. WB showed similar expression levels of NFKB, TNF-α, IFN-γ, TLR4, TGF-β and CXCL1 in Str (B) and SNc (C) between WT and LRRK2^{R1627P} or LRRK2$^{-/-}$ rats at different ages (N=4).

© 2024. Yang Q et al. Published online at http://www.aginganddisease.org/EN/10.14336/AD.2024.0883

SUPPLEMENTARY DATA

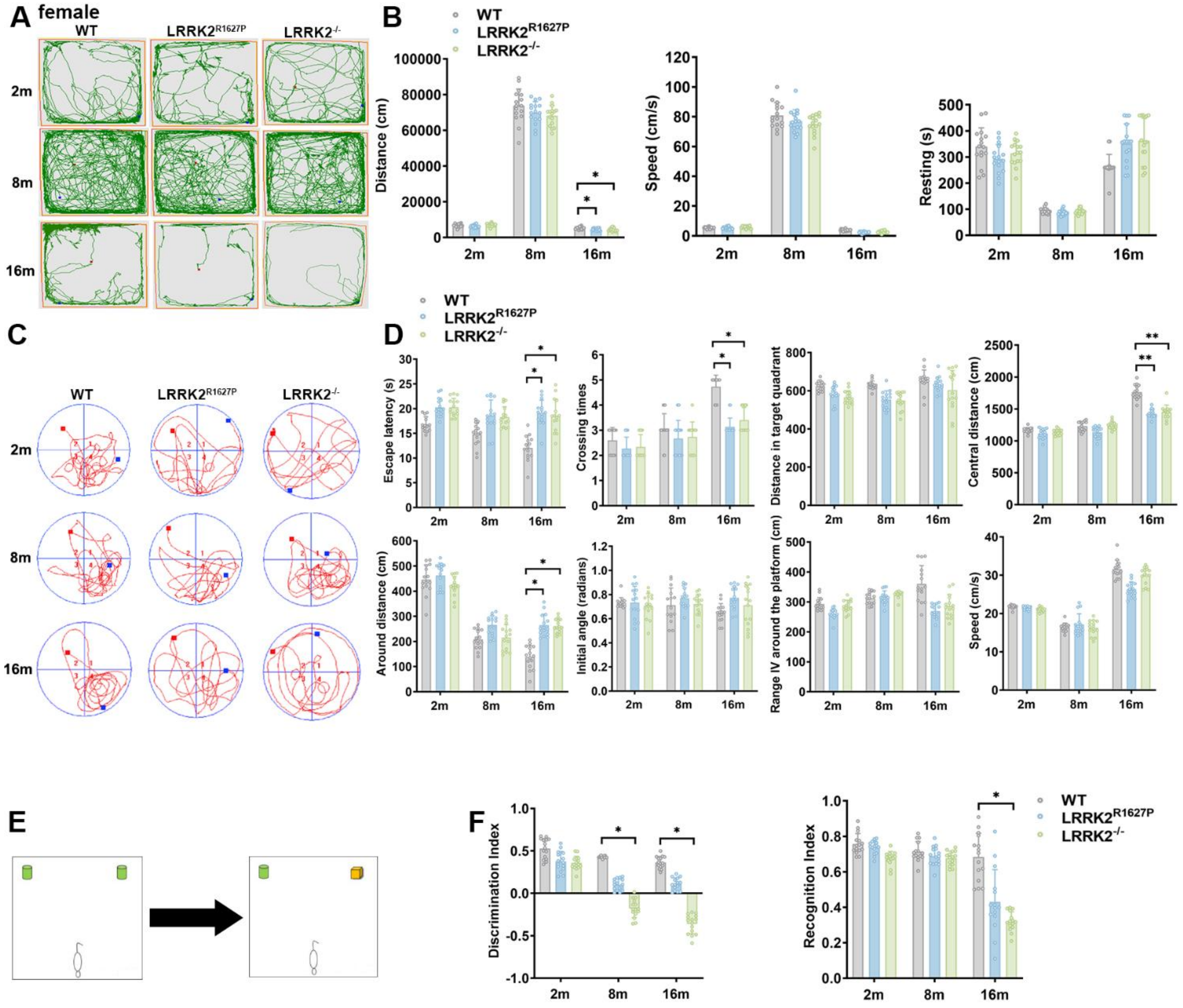

Supplementary Figure 6. Behavioral analysis of LRRK2^{R1627P} and LRRK2$^{-/-}$ female rats. (A/B) Open field assay was used to detect the exploratory behavior of WT, LRRK2^{R1627P} and LRRK2$^{-/-}$ rats at different ages (N=16). (C/D) Water maze assay was used to detect the exploratory behavior of WT, LRRK2^{R1627P} and LRRK2$^{-/-}$ rats at different ages. (E/F) The novel object recognition assay was used to detect the exploratory behavior of WT, LRRK2^{R1627P} and LRRK2$^{-/-}$ rats at different ages (N=15). Data represent mean ± SD. *P<0.05, **P<0.01.

© 2024. Yang Q et al. Published online at http://www.aginganddisease.org/EN/10.14336/AD.2024.0883

SUPPLEMENTARY DATA

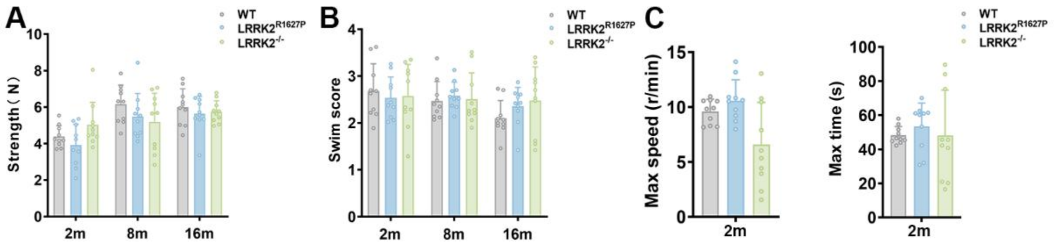

Supplementary Figure 7. LRRK2^{R1627P} and LRRK2$^{-/-}$ rats exhibit normal motor behavior. (A) LRRK2^{R1627P} and LRRK2$^{-/-}$ rats showed normal forelimb grip at different ages (N=10). (B) LRRK2^{R1627P} and LRRK2$^{-/-}$ rats showed normal swimming coordination at different ages (N=10). (C) LRRK2^{R1627P} and LRRK2$^{-/-}$ rats showed normal balance at 2-month (N=10). Data represent mean ± SD.

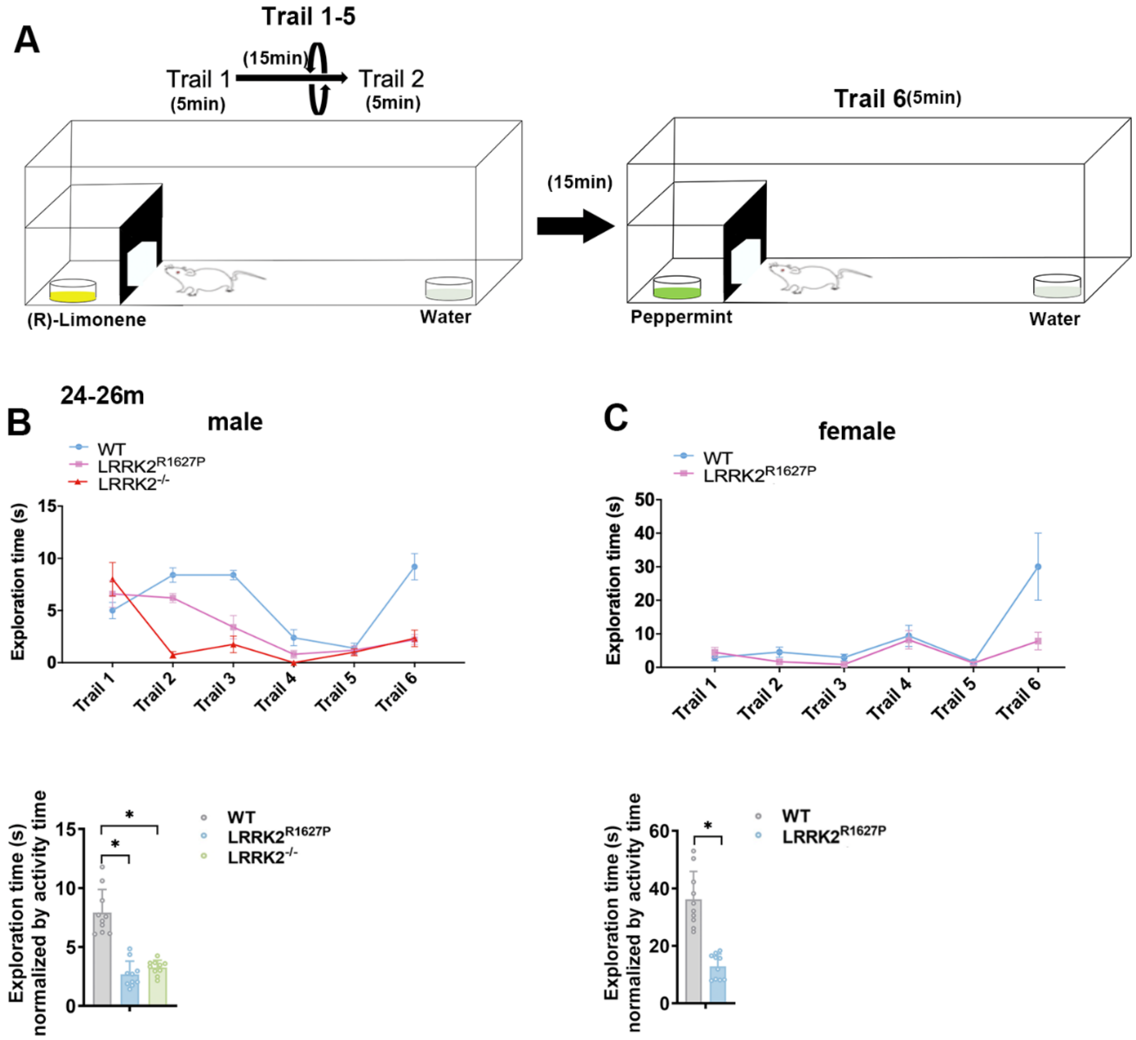

Supplementary Figure 8. Olfactory abilities are reduced in elderly LRRK2^{R1627P} and LRRK2$^{-/-}$ rats. (A) In the discrimination assay, animals are tested for their ability to discriminate a conditioned odor (lemon flavor) (yellow) and an unfamiliar odor (mint flavor) (green). (B/C) LRRK2^{R1627P} and LRRK2$^{-/-}$ rats made less correct choices and exploration time in mint flavor at 24-26-month. Data represent mean ± SD (N=10). *P<0.05.

© 2024. Yang Q et al. Published online at http://www.aginganddisease.org/EN/10.14336/AD.2024.0883

SUPPLEMENTARY DATA

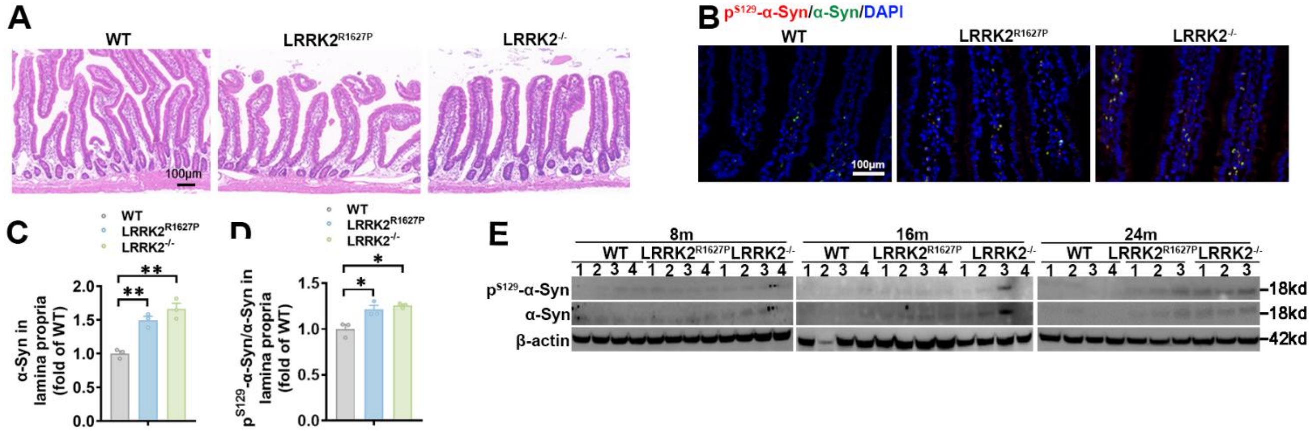

Supplementary Figure 9. The expression of total α-Syn and pS129-α-Syn were significantly increased in the small intestine of LRRK2 transgenic rats. (A) Representative HE staining images of small intestine from 8-month-old WT, LRRK2^{R1627P} and LRRK2$^{-/-}$ rats, scale bar 100 µm (N=3). (B) Double immunofluorescence staining images of α-Syn (green) and p^{S129}-α-Syn (red) in the small intestinal lamina propria from 16-month-old WT, LRRK2^{R1627P} and LRRK2$^{-/-}$ rats, scale bar 100 µm. Nuclei were stained with DAPI (blue). (C/D) The statistical analysis of α-Syn and p^{S129}-α-Syn immunofluorescence staining (N=3). (E) Western blot and relative protein expression level of α-Syn and p^{S129}-α-Syn in the small intestine at 8，16 and 24-month-old rats (N=4). Data represent mean ± SD.*P<0.05, **P<0.01.

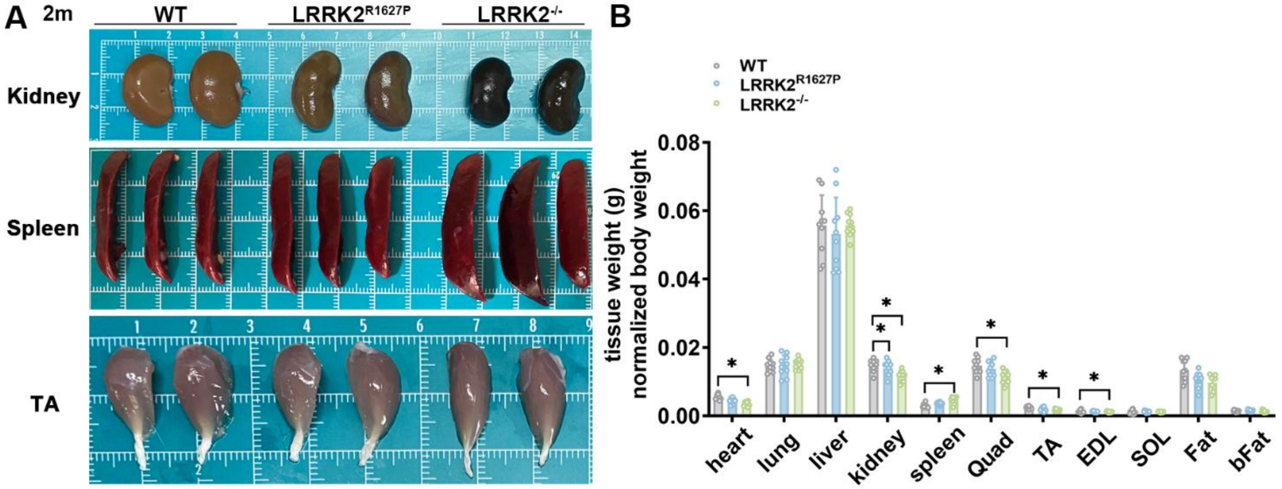

Supplementary Figure 10. Characterization of peripheral organs of LRRK2^{R1627P} and LRRK2$^{-/-}$ rats. (A) Anatomical diagrams of peripheral organs (kidney, spleen and skeletal muscle) in 2-month-old WT, LRRK2^{R1627P}, and LRRK2$^{-/-}$ rats. (B) Tissue weights of 2-month-old WT, LRRK2^{R1627P}, and LRRK2$^{-/-}$ rats (N=10). Data represent mean ± SD.*P<0.05.

© 2024. Yang Q et al. Published online at http://www.aginganddisease.org/EN/10.14336/AD.2024.0883
